# Impact of early-life feeding on local intestinal microbiota and digestive system development in piglets

**DOI:** 10.1038/s41598-021-83756-2

**Published:** 2021-02-18

**Authors:** R. Choudhury, A. Middelkoop, J. G. de Souza, L. A. van Veen, W. J. J. Gerrits, B. Kemp, J. E. Bolhuis, M. Kleerebezem

**Affiliations:** 1 Host-Microbe Interactomics Group, Department of Animal Sciences, Wageningen University and Research, P.O. Box 338, 6700 AH Wageningen, The Netherlands; 2 Adaptation Physiology Group, Department of Animal Sciences, Wageningen University and Research, P.O. Box 338, 6700 AH Wageningen, The Netherlands; 3 Animal Nutrition Group, Department of Animal Sciences, Wageningen University and Research, P.O. Box 338, 6700 AH Wageningen, The Netherlands

**Keywords:** Metagenomics, Microbiome, Animal physiology, Animal behaviour

## Abstract

Early-life gut microbial colonisation is known to influence host physiology and development, shaping its phenotype. The developing gastro-intestinal tract of neonatal piglets provides a “window of opportunity” for programming their intestinal microbiota composition and corresponding intestinal development. Here, we investigated the impact of early feeding on jejunum and colon microbiota composition, and intestinal maturation in suckling piglets. From two days of age, early-fed (EF; n = 6 litters) piglets had access to solid feed containing a mixture of fibres till weaning (day29) in addition to sow’s milk, whereas the control (CON; n = 6 litters) piglets exclusively fed on sow’s milk. Early feeding elicited a significant impact on the colon microbiota, whereas no such effect was seen in the jejunal and ileal microbiota. Quantified eating behavioural scores could significantly explain the variation in microbiota composition of EF piglets and support their classification into good, moderate, and bad eaters. Members of the Lachnospiraceae family, and the genera *Eubacterium, Prevotella*, and *Ruminococcus* were quantitatively associated with eating scores. EF piglets were found to have a decreased pH in caecum and colon, which coincided with increased short-chain fatty acid (SCFA) concentrations. Moreover, they also had increased weights and lengths of several intestinal tract segments, as well as a decreased villus-crypt ratio in jejunal mucosa and an increased abundance of proliferative cells in colon mucosa. The approaches in this study indicate that early feeding of a mixed-fibre (pre-weaning) diet changes the microbiota composition, pH, and fermentation products in the distal gut of piglets, while it also alters both macroscopic and microscopic intestinal measurements. These results exemplify the potential of early feeding to modulate intestinal development in young piglets.

## Introduction

In natural conditions, very young piglets begin to forage food items within a few days after birth^1–3^, familiarising with solid food and preparing for the weaning transition gradually over a period of 20 weeks of age approximately^4,5^. In contrast, weaning in commercial pig farms is an abrupt process that commonly takes place between 3 and 4 weeks of age, where piglets are exposed to various simultaneous stressors, including separation from their sow and littermates, new housing conditions, unknown pen-mates, and a sudden change of diet. These abrupt changes are often accompanied by a transient low feed intake, poor growth, intestinal dysbiosis and diarrhoea post-weaning, thus compromising animal health and welfare, increasing piglet mortality and causing economic losses^6–11^. Creep feeding, a method of supplementing suckling piglets with solid feed, is prevalent in modern pig farming to ease the weaning transition as well as stimulate post-weaning eating (or feed intake)^7,12^. However, traditional creep feed is highly palatable, easily digestible and mainly based on milk proteins^13,14^, which is distant from fibrous solid feed exposure in both natural and post-weaning conditions.

At the time of weaning, the gastro-intestinal tract of a young pig is still developing^10,15^ and undergoing rapid changes in gut microbiota colonisation, digestive system and immune development^11,16–18^. Importantly, the gut microbiome has been recognised to play a crucial role in overall animal health and development, especially in early-life^19–22^. The early-life microbial colonisation with potentially beneficial and diverse gut microbes can influence the maintenance of intestinal homeostasis and prevent gut dysbiosis^11,23,24^. Dietary fibres can modulate the gut microbiome, and they are widely recognised as food/feed components that influences gut health positively^25^. Notably, dietary fibres have also been implicated in gastrointestinal tract development and mucosal changes in pigs^26,27^. These fermentable fibres pass through the small intestine undigested and act as a substrate for the distal gut microbiota, stimulating microbial fermentation and short chain fatty acid(s) (SCFA(s)) production in the colon. The predominant SCFAs formed (approximately 95%) are acetic, propionic and butyric acid, although some other organic acids can be detected as well, such as lactic, succinic, isovaleric, and isobutyric acid^28^. Absorbed SCFAs can provide up to 15% of the maintenance energy requirement of growing pigs and 30% in gestating sows^29^. However, special attention is commonly given to butyric acid because it serves as a major source of energy for colonic epithelial cells, and has been proposed to exert several (additional) effects that are considered pivotal in establishment and maintenance of homeostasis in the colon mucosa, including colonocyte growth and proliferation^17,30–34^. The SCFAs, particularly butyric acid, can modulate the expression of genes involved in gut motility, host defence and inflammatory responses, contributing to formation and protection of intestinal barrier as well as stimulating differentiation and regulation of T cells^35,36^. Although the exact mechanisms by which microbial SCFAs influence mucosal physiology remain to be resolved, a few effects of SCFAs and the underlying mechanisms have been revealed, including the function of SCFAs as ligands for G protein-coupled receptor pair GPR41, GPR43 in epithelial or immune cells, and their inhibition of histone deacetylases (HDAC) activity^17,37^.

Although previous studies have characterised porcine gut microbiota in relation to dietary fibre intervention, most of them have focussed on the post-weaning period, assessing the impact on weaned piglets^38–42^. Currently, a handful of studies have assessed how early-life (pre-weaning) feeding might influence the gut microbiome^43–46^ and contribute to the intestinal development of neonatal piglets^27,47–49^. In our previous study^50^, we established that early feeding with mixed fibrous feed from 2 days of age, accelerates pre-weaning microbiota colonisation patterns towards those that resemble a typical post-weaning microbiome. In the present study, we evaluated the impact of early-life feeding strategy on the intestinal microbiota composition in different regions of the intestinal tract, and investigate its consequences for intestinal development and maturation. We hypothesised that the pre-weaning consumption of solid mixed-fibre feed would result in an increased level of undigested substrate in the colon, and investigated its impact on local microbiota composition, SCFA production, macroscopic development of the digestive system, as well as its microscopic consequences on mucosal morphology. Suckling piglets show large variation in solid feed intake before weaning [between^51^ and within litters^52^], and therefore we exploited the quantified variation in eating behaviour of piglets to assess the impact of early feeding at an individual piglet level.

## Results

### Gut microbiome composition in different intestinal segments

We assessed the microbiota composition in the jejunum, ileum, and colon of 28 piglets sacrificed at the end of the pre-weaning phase (day 29). Illumina Miseq 16S rRNA gene sequencing of the V3–V4 region generated 1,211,527 number of reads after quality filtering, with a mean sample depth of 16,596 ± 3,844 reads.

**Figure 1:**
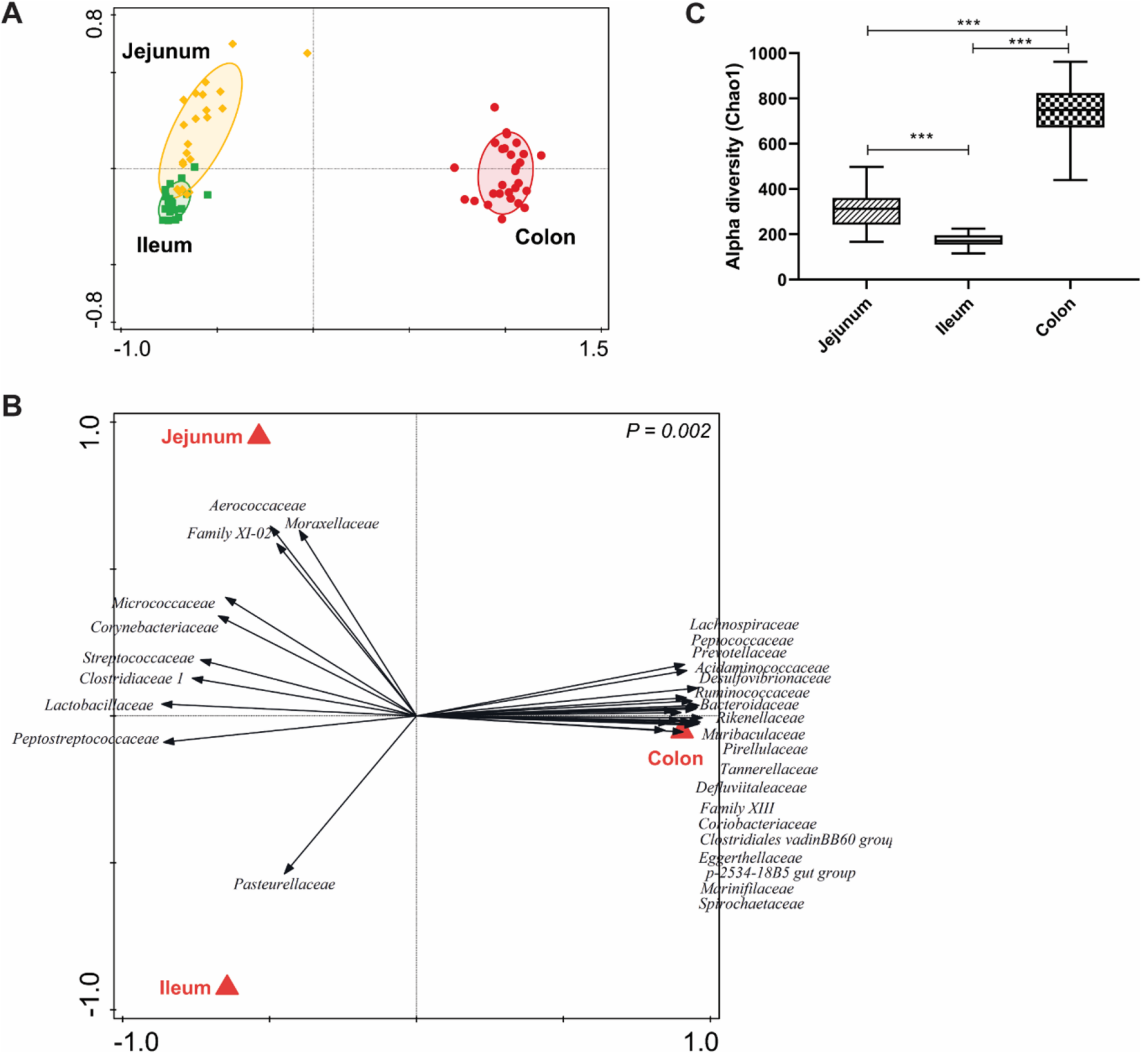
Microbiota composition along the intestinal tract. (**A**) Principal component analysis of jejunum, ileum and colon microbiota (PC1 = 66.66%, PC2 = 4.76%) at genus level. (**B**) Redundancy analysis (explained variation = 67.8% *P* = 0.002) of intestinal location with associated microbial groups at family level. Microbial groups visualized in this figure have a minimum fit value of at least 40% on the horizontal axis and a response score > 0.63 and > 0.80 for jejunum/ileum and colon, respectively. Specific microbial families which are differentially abundant in jejunum/ileum were enforced in this graph). (**C**) Alpha diversity (Chao1 bias corrected) comparison among jejunum, ileum and colon. Significant differences between groups were assessed by student t test or Mann–Whitney U test (***: *P* < 0.001).

Prominent (intestinal) location-specific differences in microbiota composition were observed. Principal component analysis showed two distinct clusters (Fig. 1A), reflecting different early-life microbial colonisers in the small and large intestine. For example, microbial families like Lactobacillaceae, Peptostreptococcaceae, Clostridiaceae 1 were found to be dominant in the small intestinal (jejunal and ileal) samples whereas Rikenellaceae, Prevotellaceae, Ruminococcaceae, Lachnospiraceae were more abundant in the large intestine (Fig. 1B; Supplementary Fig. 2A). This was further demonstrated by microbiota compositional (redundancy) analysis as a function of ‘intestinal location’ which identified dominant microbial groups associated with each intestinal segment (Fig. 1B). The small difference between jejunum and ileum microbiota was recognised by the limited explained variation (4.76%) encompassed by the second principal component (PC2), as compared to the first principal component (PC1; explained variation 66.66%), which separates small and large intestinal microbiota (Fig. 1A). The large intestine had microbiota with a higher microbial richness (Fig. 1C) and evenness (Supplementary Fig. 3A) compared to the small intestine and clustered separately when assessed by Bray Curtis distance (Supplementary Fig. 3A; PERMANOVA, *P* < 0.0001). Comparing jejunal and ileal microbiota, Aerococcaceae, Fusobacteriaceae, Moraxellaceae were found to be more abundant in jejunum, while only Pasteurellaceae was found to be more abundant in ileum (Supplementary Fig. 2B), which corroborated the redundancy analyses for intestinal location (Fig. 1B). Remarkably, we observed significant differences in alpha and beta diversity between jejunal and ileal microbiota (Supplementary Fig. 3B). Jejunum microbiota was found to have a higher richness (Fig. 1C) compared to ileum, although there was no difference observed in evenness (Supplementary Fig. 3B). These findings established prominent differences in microbiota composition in small and large intestinal locations, and underpin the high relatedness between the microbiota of the jejunum and ileum regions of the small intestine, which was also supported by the partial separation of jejunal and ileal samples in hierarchical clustering (Supplementary Fig. 4).

**Figure 2:**
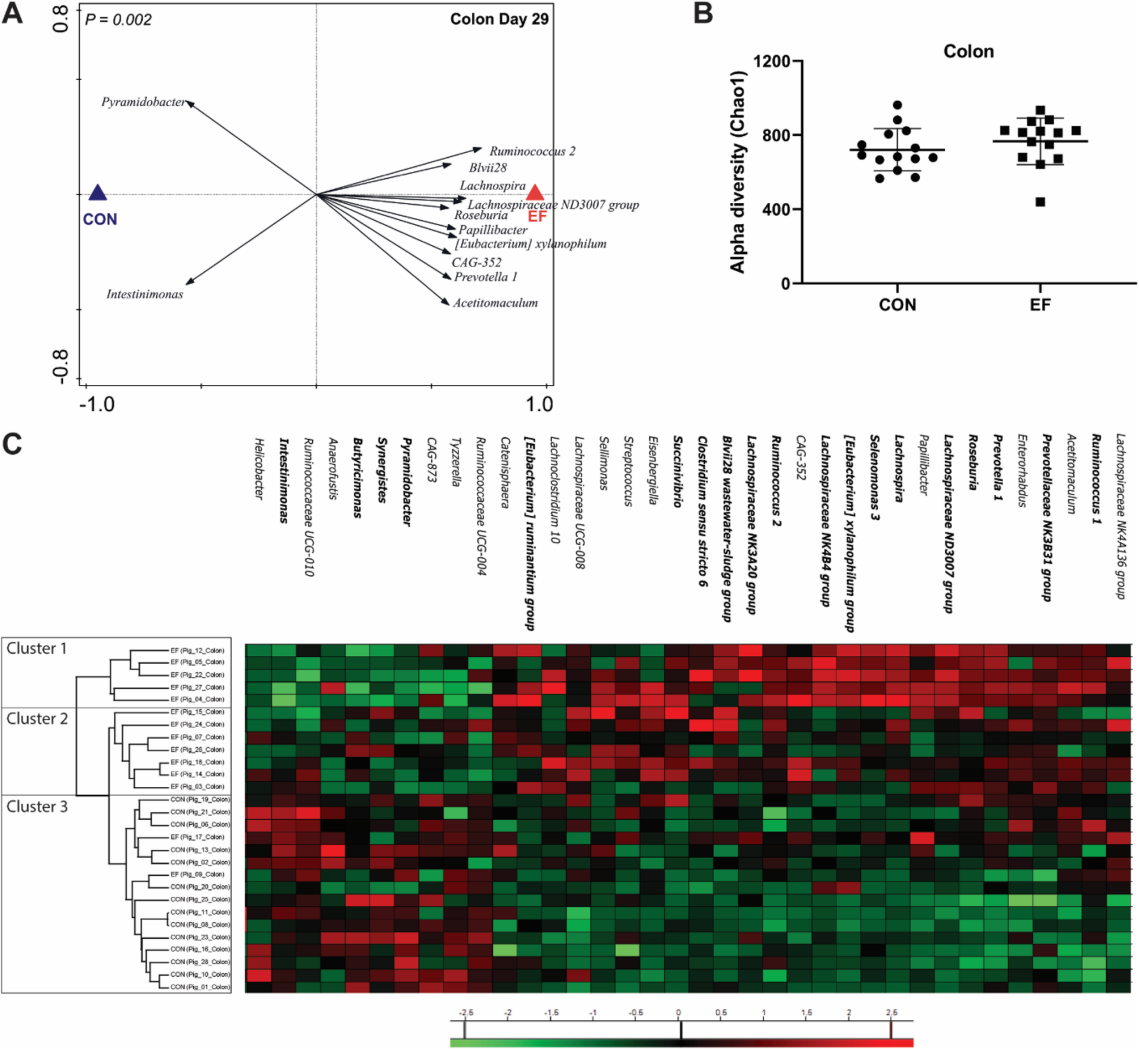
Colonic microbiota composition in early-fed (EF) and control (CON) group. (**A**) Redundancy analysis at genus level (PC1 = 8.75%, PC2 = 16.12%; *P* = 0.002) with associated microbial groups shown (minimum fit value of at least 30% and > 0.55 response score on horizontal axis). (**B**) Alpha diversity (Chao1 bias corrected) comparison between the two groups. (**C**) Heat map showing relative abundance of discriminative bacterial genera (≥ 0.40 response score in x axis) as found in redundancy analysis. RDA identified microbes that were also detected in a previous study with similar design^50^ are shown in bold.

**Figure 3:**
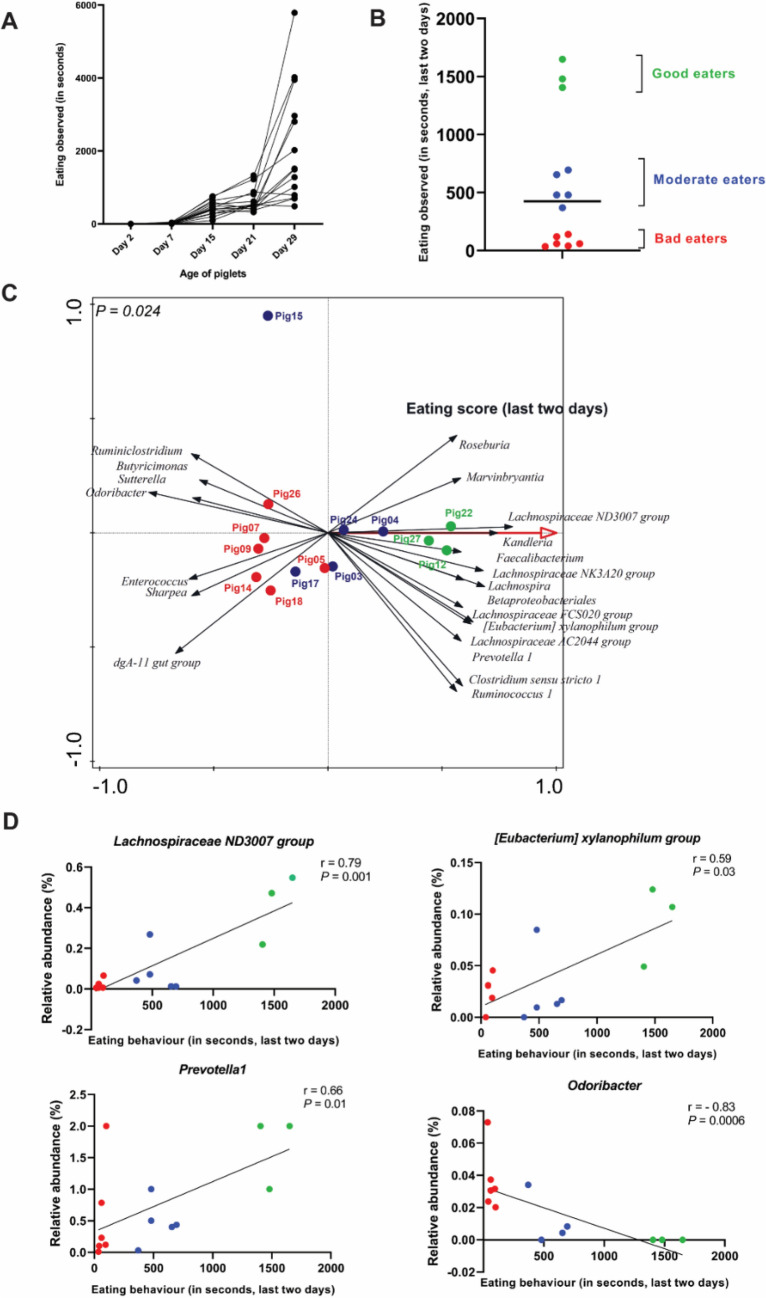
Classification of early-fed (EF) piglets into good (green), moderate (blue) and bad (red) eaters. (**A**) Individual piglet’s eating behaviour quantification (14 EF piglets; total eating seconds per week) for four weeks pre-weaning by video observation. (**B**) Good (> 2 × median; green), moderate (between 0.8 × and 2 × median; blue) and bad (below 0.8 × median; red) eaters, grouping based on eating observed in the “last two days” before weaning. (**C**) Redundancy analysis based on eating scores from “last two days” before weaning (explained variation = 5.42%, *P* = 0.024), establishing the microbiota discrimination between the “good”, “moderate” and “bad” classification within the EF piglets (minimum fit value of at least 30% and > 0.55 response score on horizontal axis). (**D**) Spearman correlation of individual microbial genera with the eating score from “last two days”. *Lachnospiraceae ND3007, Eubacterium xylanophilum, Prevotella 1 and Odoribacter* were identified in redundancy analysis.

**Figure 4:**
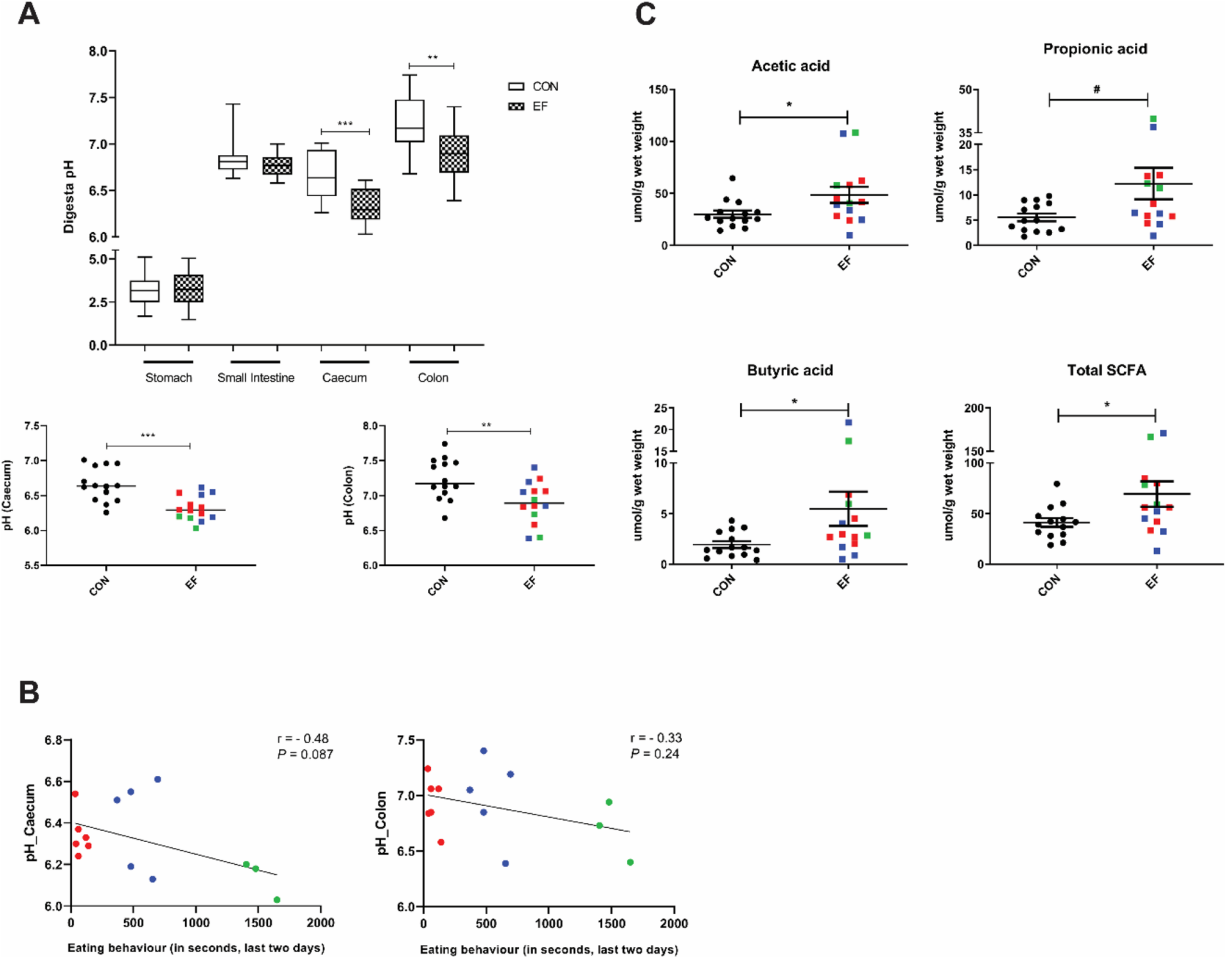
Impact of early feeding on pH and short chain fatty acids (SCFAs). (**A**) Digesta pH in different parts of the GIT for EF and CON and an expansion of the two segments where significant difference in pH was detected, illustrating the distribution of individual piglets in both groups (using the EF group classification of good (green), moderate (blue) and bad (red) eaters; see Fig. 2B). (**B**) Correlation of pH caecum/colon of individual EF piglets with the quantified eating score from last two days (Caecum: r =  − 0.48, *P* = 0.087; Colon: *r* =  − 0.33, *P* = 0.24). (**C**) Group level comparison for colonic SCFA concentration (µmol/g wet weight of digesta) in piglets. Significant differences between groups were assessed by student t test or Mann–Whitney U test (*: *P* < 0.05; **: *P* < 0.01; ***: *P* < 0.001). EF = early-fed group; CON = control group. Green = good eaters; Blue = moderate eaters; Red = bad eaters.

### Effect of early feeding on local intestinal microbiota

One of the main objectives of this study was to gain insight into the impact of early-life feeding on jejunal, ileal and colon microbiota. We did not find any impact on small intestinal (jejunal and ileal) microbiome composition or diversity due to early (pre-weaning) feeding of piglets (Supplementary Fig. 5). In contrast, the colon microbiome was found to be significantly altered due to early feeding, and RDA analyses identified several microbial groups (such as *Ruminococcus 2, Lachnospira, Lachnospiraceae group ND3007, Roseburia, Papillibacter, Eubacterium, Prevotella 1)* associated with this difference (Fig. 2A). Notably, some of these microbes (Fig. 2C; marked in bold) were also detected to be enriched in rectal swab samples taken pre-weaning from early-fed piglets in a previous study^50^. Those microbial groups represent typical post-weaning associated microbes, demonstrating that early feeding with a fibrous diet accelerates the ‘maturation’ of the microbiota towards a post-weaning composition. Due to the absence of post-weaning microbiota samples in this study (piglets sacrificed before weaning), this correlation with the post-weaning microbiota cannot be confirmed, but the association of the same microbial groups with early feeding supports the similarity of microbiota impact. To further evaluate the impact of early feeding on microbiota composition, beta diversity was assessed by Bray Curtis distance, which revealed significant dissimilarity between the EF and CON groups (PERMANOVA, *P* = 0.04; data not shown), although no significant difference was observed in alpha diversity (Fig. 2B).

**Figure 5:**
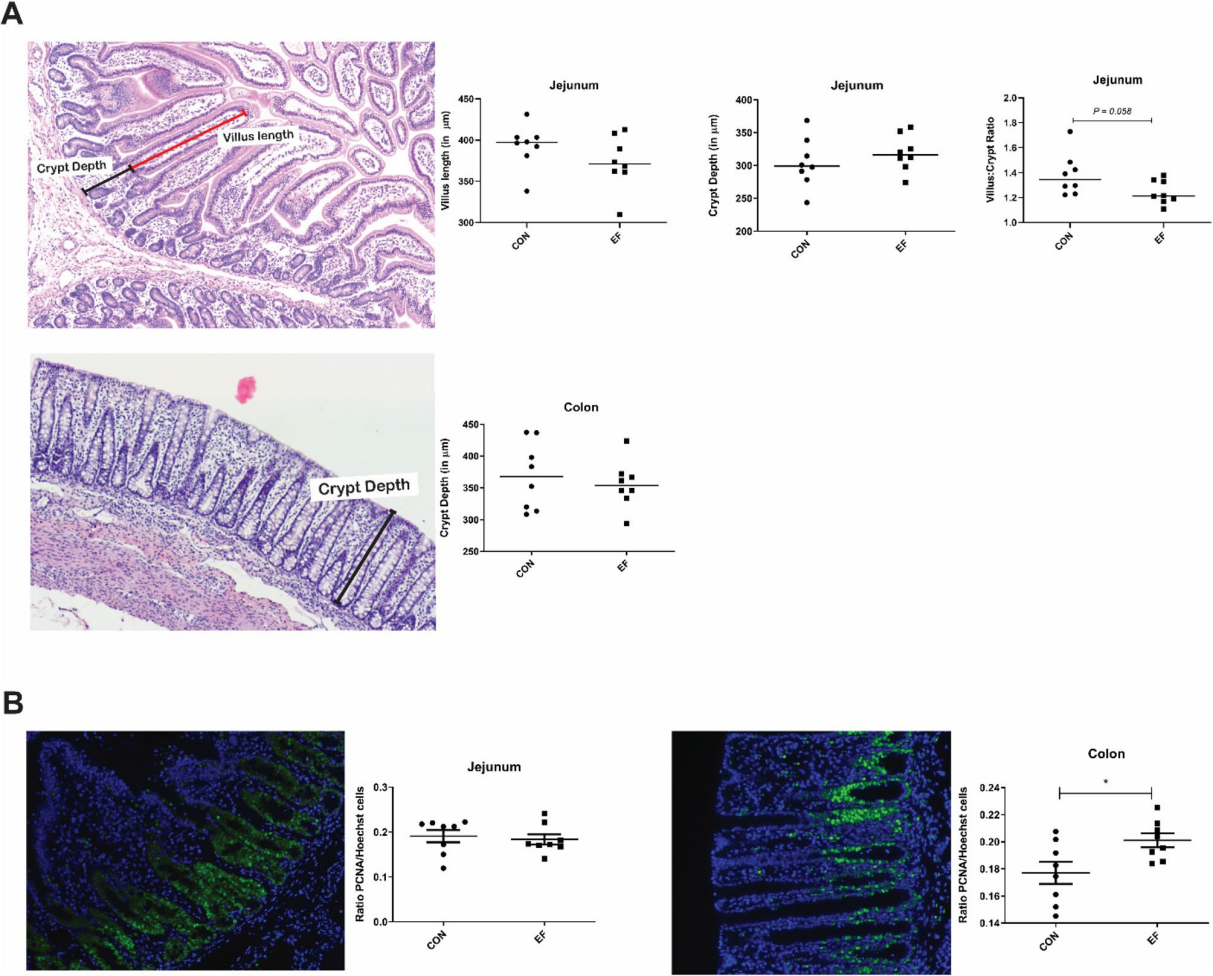
Effects of early feeding on (**A**) intestinal morphometry and (**B**) PCNA^+^ proliferating cells in jejunum and colon (representative image) at weaning (day29). Hoechst and PCNA positive cells are represented in blue- and green-coloured cells respectively. EF = early-fed group; CON = control group. Differences between groups were assessed by student t test or Mann–Whitney U test (*, *P* < 0.05). The bars in these images illustrate the measured parameters. Representative images of CON and EF groups can be found in Supplementary Fig. 11.

To assess the impact of early feeding at individual animal level, hierarchical clustering of all samples was performed based on EF/CON group-associated microbes (detected in RDA analyses; Fig. 2A). Partial separation of EF and CON piglets was observed at individual animal level (Fig. 2C). Overall, the hierarchical clustering divided the piglets into three main clusters: (1) five EF piglets clustering clearly separate, (2) seven EF piglets clustering together but less distant from the next cluster (3) encompassing all the CON piglets and the remaining two EF piglets. The genera comprising of *Prevotella 1, Roseburia, Lachnospiraceae ND3007 group, Lachnospira, Selenomonas 3, Roseburia, Eubacterium xylanophilum, CAG-352 and Ruminococcus 2* were found to be most abundant in the first cluster whereas the second cluster had a more variable abundance of these groups. Cluster 3 was characterised by higher abundance of other microbial groups, including *Ruminococcaceae (UGC-004, UGC010), Tyzzerella, CAG-873, Pyramidobacter, Synergistes, Butyricimonas and Intestimonas*. Intriguingly, some of these microbes such as *Butyricimonas, Pyramidobacter, Intestimonas* and *Synergistes* were also found associated with the CON group in our previous study^50^.

### Quantitative estimate of eating behaviour

To estimate the eating time per individual piglet, video recordings of the six EF litters (12 h per day) were observed and eating bouts were recorded during the four-week pre-weaning period. The eating scores were employed as a quantitative indication of eating. The eating behaviour of the EF piglets gradually increased over time, reaching the highest eating score in the last week pre-weaning (Fig. 3A), which is similar to our previously reported observations^50^. However, the quantification of eating behaviour in the present study was more variable and on average lower as compared to our previous study of a similar design (Supplementary Fig. 6). Nevertheless, the substantial variation in the estimated eating among EF piglets in the present study provides the opportunity to evaluate the relationship between the individualised quantification of eating behaviour and the piglet-specific microbiota composition and intestinal measurements.

Previously it was shown that eating quantification observed shortly before (rectal swab) microbiota sampling is strongly and quantitatively related to the microbiota changes, that are driven by consumption of the fibre-enriched feed^50^. Analogously, in this study, we employed the summed eating scores of the last two days prior to sacrifice to investigate their relationship with the microbiome signatures observed in individual EF piglets. Based on the eating scores in the last two days, we can classify individual EF piglets into good (> 2 times the median; green), moderate (between 0.8 and 2 times the median; blue) and bad (below 0.8 times the median; red) eaters (Fig. 3B). The RDA analyses show that these individual eating scores (cumulative eating scores from the “last two days”) could significantly explain the colon microbiota composition (Fig. 3C). Notably, all periodical eating scores (“Total seconds”, “Last week”, “Last two days” and “Last day”) were significantly and positively correlated with each other (Supplementary Fig. 9), and were all significantly reflected by the microbiota RDA scores (Supplementary Figs. 9 and 10). Moreover, the microbiota composition supported the classification of EF piglets into good (green), moderate (blue) and bad (red) eaters on the basis of discriminatory microbial groups that were either more abundant in good eaters (e.g., *Lachnospiraceae ND3007 group, Lachnospiraceae NK3A20 group, Kandleria, Eubacterium xylanophilum, Lachnospira, Prevotella1, Faecalibacterium, Roseburia*) or more abundant in the bad eaters (e.g., *Odoribacter, Butyricimonas*). However, since the overall group of EF piglets in this study includes a substantial number of bad eaters, significant correlations were observed between only a few individual microbial groups and eating scores (Fig. 3D, Supplementary Fig. 7). Nevertheless, the individualised eating behaviour substantiated the partial separation of EF piglets in hierarchical clustering (Fig. 2C) where some EF piglets (classified as bad eaters) were clustering closely to the control piglets. Taken together, these findings underpin the microbiota compositional changes in response to individualised eating behaviour.

### Changes in digesta pH and SCFA concentration

To assess the intestinal fermentation as a result of early feeding, the intraluminal pH in different segments of the intestinal tract and the concentration of short chain fatty acids (SCFAs; i.e., succinic-, lactic-, acetic, propionic- and butyric-acid) in the colon content were determined. The digesta pH of both caecum and colon significantly decreased due to early feeding of piglets (*P* < 0.05), whereas no differences in stomach or small intestinal digesta were detected (Fig. 4A). Subsequently, the relationship between eating scores and digesta pH in individual EF piglets was investigated, revealing that caecum pH tended to be negatively correlated with eating behaviour, whereas no such association was observed for the colon (Fig. 4B). Nevertheless, the levels of specific colonic SCFA were significantly impacted by early feeding, and relative to the CON group, concentrations of acetic acid, butyric acid and total SCFAs were significantly higher whereas propionic acid tended to be higher (*P* = 0.06) in the EF group (Fig. 4C), indicative of increased carbohydrate fermentation in the distal gut. As anticipated, caecum and colon pH negatively correlated with SCFAs acetic acid, propionic acid and butyric acid. In contrast, no differences were observed in lactic- and succinic-acid between the CON and EF piglets (Supplementary Fig. 8). Notably, SCFA (acetic-, propionic- and butyric acid) concentrations in individual piglets were found to be significantly correlating with each other (Supplementary Fig. 9). However, the distribution of the differential eating classifications (good, moderate and bad eaters) did not appear to be significantly related to the levels of SCFAs observed, although a trend (*P* = 0.08) of positive correlation was observed between propionic acid and eating scores (Supplementary Figs. 9 and 10). Remarkably, the caecal pH appeared to be significantly reflected by the levels of acetic, propionic and butyric acid (and total SCFAs) measured in the colon, whereas colonic pH values only correlated with the level of butyric acid (Supplementary Fig. 9).

### Digestive organ measurements (macroscopic)

Macroscopic measurements such as digestive organ weight and lengths were determined to check the effect of early feeding on the intestinal development of piglets at weaning (Table 1). Notably, there was no significant difference in the pre-weaning body weight development between the groups (Supplementary Fig. 1B; for details see^53^). Analogously, EF and CON piglets did not differ in organ weights of the adrenal gland, gallbladder, spleen, liver, stomach, and caecum. However, EF piglets tended to have a heavier pancreas (*P* = 0.05) compared to CON piglets. The small intestine (*P* = 0.096) and the total intestinal tract (*P* = 0.05) showed a tendency to be longer in EF piglets, while the weights of the small intestine and the total intestinal tract were higher (*P* < 0.05), both with (full) and without (empty) digesta in EF piglets. Further, the large intestine (including caecum and colon) was significantly longer (*P* < 0.05) and tended to be heavier with digesta (*P* = 0.08) in EF piglets.

**Table 1:** Intestinal weights of early-fed (EF) and control (CON) piglets at weaning (d29), mean ± SEM. *P*-values are based on linear mixed model using body weight at sacrifice as covariate.

Item	CON	EF	*P*-value
Body weight, kg	8.2 ± 0.3	8.5 ± 0.4	0.57
**Organ weight, g**			
Adrenal gland	0.5 ± 0.02	0.5 ± 0.03	0.70
Pancreas	13.5 ± 0.7	15.5 ± 0.8	0.05
Spleen	19.4 ± 1.0	19.4 ± 1.0	0.70
Liver	213.6 ± 7.2	219.4 ± 11.0	0.79
Gallbladder	3.0 ± 0.4	2.5 ± 0.3	0.39
**Intestine weight, g**			
Stomach, full	146.2 ± 10.7	160.7 ± 11.0	0.47
Stomach, empty	43.2 ± 1.6	44.7 ± 1.7	0.76
Small intestine, full	421.7 ± 17.8	482.5 ± 31.3	0.02
Small intestine, empty	328.5 ± 10.5	370.3 ± 20.3	0.03
Cecum, full	47.9 ± 2.5	51.0 ± 3.5	0.67
Cecum, empty	18.8 ± 1.1	19 ± 0.9	0.89
Colon, full	80.4 ± 4.2	96.8 ± 7.5	0.08
Colon, empty	47.2 ± 2.4	50.3 ± 3	0.54
Total gastro-intestinal tract, full	696.2 ± 27.3	790.9 ± 45.9	0.02
Total gastro-intestinal tract, empty	437.7 ± 14.3	484.3 ± 23	0.03
**Intestine length, m**			
Small intestine	7.8 ± 0.2	8.3 ± 0.2	0.096
Large intestine^1^	1.2 ± 0.02	1.3 ± 0.03	0.046
Total gastro-intestinal tract	9 ± 0.2	9.6 ± 0.2	0.05

The intestinal segment lengths showed a significant positive correlation with each other, however, organ and intestinal weights did not show such correlation (Supplementary Fig. 9). Notably, the weight of the small intestine (without digesta; empty) as well as the whole gastrointestinal tract (with or without digesta; full or empty) significantly correlated (*P* = 0.01; Supplementary Figs. 9 and 10) with the eating scores (“total seconds”), whereas small intestine (with digesta; full) tended to show the same correlation (*P* = 0.06; Supplementary Figs. 9 and 10). Taken together, these results indicate that early eating has (moderate) effects on some macroscopic measurements of the digestive system (Table 1), but not all these effects appear to significantly correlate with the quantified estimate of eating.

### Mucosal morphometry and epithelial proliferation in jejunum and colon

Intestinal morphometry (microscopic) measurements were assessed using histological staining (n = 8; randomly selected piglets from each treatment group) of jejunum and colon mucosa. No significant alteration was observed in the villus length or crypt depth in the jejunal mucosa, nor in the colonic crypt depth of tissue samples from EF compared to CON group (Fig. 5; Supplementary Fig. 11). However, due to a combination of modestly increased villus length and decreased crypt depth in jejunal mucosa, we observed a tendency for a reduced villus length : crypt depth ratio (V:C ratio) in the EF compared to the CON animals (*P* = 0.06). However, no quantitative association was found between these mucosal morphometric measurements and eating scores (Supplementary Figs. 9 and 12), although it should be noted that the animals selected for this analysis were not evenly distributed over the good-, moderate- and bad-eaters classification within the EF group. For example, the inclusion of only a single piglet of the “good eater” group may have led to an underestimation of the relatedness of the eating scores and the mucosal morphometric differences.

Epithelial proliferation was evaluated using immuno-histochemistry in combination with a standardised quantification pipeline in jejunum and colon mucosa. To determine the relative amount of proliferative epithelial cells in the jejunum and colon mucosa samples, tissues were stained with nuclei stain PCNA and Hoechst (n = 8; randomly selected piglets from each treatment group). There was an increase in the number of colonic proliferating cells in EF group compared to CON (ratio of PCNA:Hoechst positive cells), however, no such difference was observed in jejunal tissue (Fig. 5). Notably, eating scores (based on last two days and last day) correlated with proliferating cells in jejunum (Supplementary Figs. 9 and 12), although the effect size is very low (Cohen’s d ≤ 0.2) indicative of substantial individual variation, raising questions about the reliability of this observation.

## Discussion

Early-life conditions are known to have a key influence on the developing gut microbial ecosystem and have been shown to have long lasting consequences for the microbiota as well as the host^10,54,55^. The present study was aimed to investigate whether early-life feeding (pre-weaning access to a mixed-fibre feed) has an impact on the intestinal microbiota composition and digestive system maturation at weaning. The hypothesis was that early feeding (of mixed-fibre feed) would modulate the microbiota composition in the intestine of piglets and would support digestive system development. Our results show that early feeding impacts the intestinal microbiota and its metabolism (short chain fatty acids), as well as digestive system development, determined at both macroscopic and microscopic level. Importantly, we observed the microbiota compositional changes only in the colon and their magnitude responds proportional to the individual piglet’s quantified eating behaviour during the pre-weaning period. In contrast, many of the macro- or microscopic digestive system changes associated with early feeding did not appear to be related with the eating quantification, suggesting that these changes could be driven by feed exposure (and/or its associated intestinal microbiota) per se, rather than the amount of feed consumed.

Early feeding did not appear to elicit significant changes in the small intestinal microbiota which was very different in composition and diversity as compared to the colon microbiota (irrespective of the intervention). Substantial differences between the microbiota in these different regions are in agreement with earlier studies^56–59^. Our analyses detected a significant difference in richness (Chao1) between jejunal and ileal microbiota samples. However, previous studies have reported conflicting conclusions related to similar analyses^40,56–58,60^, which may be due to a high dynamics of the small intestinal microbiota that was suggested to be driven by many (nutritional and environmental) factors^61,62^ that differ within and between studies and thereby intrinsically prohibit consistent conclusions.

The impact of early feeding (of fibrous feed) on the colon microbiota is in accordance with previous studies^43,44,63^, and corroborates the impact of dietary fibres on the distal regions of the intestine. Various studies have reported the influence of fibres on the microbiota composition, mostly focussing on weaned or growing pigs^38–41,64^. In the present study, a customised mixed-fibre feed was formulated especially for suckling piglets, with inclusion of both soluble (GOS, inulin) and insoluble (oat hulls, sugarbeet pulp, resistant starch) dietary fibres. In line with our previously described observations^50^, early feeding with this diet elicited higher relative abundances of fibrolytic and/or butyrate-producing bacterial groups, like *Ruminococcus, Lachnospira, Roseburia, Eubacterium,* and *Prevotella*, which reflect the accelerated pre-weaning microbiota development towards a “post-weaning-resembling” microbial-ecosystem.

Consistent with previous studies^52,65,66^, a large variation in eating behaviour was observed among the EF piglets. In addition, the eating behaviour of the EF piglets in the present study, may have been compromised by a diarrhoeic episode during the third week (spreading between day 16 and 24) in almost all litters, the cause of which is unknown. Bruininx and coworkers^67^ concluded that there was considerable within-litter variation in creep-feed intake, and designated piglets as good, moderate and non-eaters based on the colour of their faeces using chromium oxide as a marker. Here we used a similar classification system on eating behaviour video-observation scores and it should be noted that a relatively large proportion of the dissected piglets was classified as bad eaters (42%), which is substantially more than in our previous study^50^ that employed a similar design. The higher degree of feed-intake variation in this study, was exploited to correlate the eating scores with the microbiota changes per individual piglet, demonstrating that the eating time is strongly reflected in the magnitude of the colon microbiota changes. Importantly, this reflection appeared independent of the eating score (time-period) employed, supporting the robustness of the observation that the eating time is a key-driver of the microbiota adaptation. This is in good agreement with our previous study^50^, where it was shown that the microbiota analysed in rectal swabs at different stages during the pre-weaning period accurately reflects the eating behaviour quantification in piglets.

It is well established that dietary fibre reaches the large intestine, due to their indigestibility by the host digestive enzymes that are released in the proximal small intestine. In the colon, these fibres are fermented by the colonic microbiota into short chain fatty acids (SCFAs)^25^. Prior studies have shown elevated levels of SCFAs after fibrous diet intake in piglets, which was associated with lower pH values in distal gut^27,47,49^. Analogously, in the present study EF piglets had higher levels of the canonical SCFAs (acetic, propionic and butyric acid) in their colon, which was reflected by a lower pH of both caecal and colonic digesta. Notably, although eating time tended to quantitatively correlate with caecal pH in our study, it did not associate with colonic pH or colonic SCFA levels. A possible explanation for this might be that luminal SCFAs production and their absorption by the colonic epithelium is highly dynamic^68^, whereby colonic SCFA levels poorly represent the flux of SCFA production and absorption^69^. Thereby, the caecum, where the fermentative capacity of the microbiota is considered to be at its maximum^70^, and where mucosal absorption rates of the luminal SCFAs may be lower as compared to the colon, might have provided a more appropriate intestinal region to assess SCFA concentrations changes in relation to dietary intake, compared to the colon.

It is important to note that in this study we have employed a customised (fibrous) feed composition for suckling piglets which is distant from the traditional, milk-based creep feed composition. Since we do not have a “traditional creep feed” treatment group in the experiment, it will be difficult and speculative to make comparisons with our study. Keeping in mind that diet is one of the major determinants of the microbiome, the anticipation will be that the (non-fibrous, highly digestible) traditional creep feed will probably have a substantially lower impact on microbiota. Further studies would be necessary to disentangle the impact of early-life feeding and the effect of fibrous diet, as they might be different.

Increased weight of digestive organs, including the weight of the intestine itself as well as its length have been associated with solid food consumption^27,71,72^. Our study reached related conclusions, where early feeding (with mixed-fibre feed) was associated with (relatively modest, but significant) changes in macroscopic digestive organ measurements, including a heavier small intestine (empty and full) and complete gastro-intestinal tract (empty and full), as well as an extended large intestine and complete gastrointestinal tract length. However, only a few of these changes appeared to have an association with the quantified feed-intake estimates within the early-feeding group, although the relatively small number of animals may have obscured such relationships, and to definitely disqualify them would require the analysis of a larger amount of EF animals. For example, eating behaviour was associated with the weights of the small intestine and total gastro-intestinal tract (with or without digesta). Increase in gut fill may be due to the typical water-retention activity and ‘bulking agent’ capacities of the (insoluble) fibres^73,74^ that are present in pre-weaning diet^26^. These results suggest that early feeding of a fibre-enriched feed stimulates expansion of the intestinal size as well as digesta. This notion was further supported by a significant positive correlation between the changes in microbiota (microbiota scores RDA), colonic SCFA concentrations and the weight of the small intestine, colon and gastrointestinal tract with or without its digesta, indicating their interrelatedness. Intriguingly, some measurements significantly correlated, for example, SCFAs associated with a few macroscopic measurements such as weight of gall bladder and colon (with digesta). However, the correlations were mainly depending on EF (good and moderate) piglets with relatively extreme SCFA-level values compared to other piglets, which is most likely a chance event in the intestinal dynamics of SCFA production and absorption. Thereby the limited numbers of animals in this study and the observation that these observations are largely determined by a small proportion of these animals may indicate that these conclusions are biologically less reliable and would require further studies that include larger numbers of animals.

Presence of luminal nutrients in the gut can cause changes to the structure and function of the intestinal mucosa^11,75^. Feed intake has been positively associated with adaptation of mucosal architecture, i.e., altered villus length or V:C ratio, which has in particular been reported in relation to post-weaning intestinal adaptations^76–79^. However, these effects have not been unambiguously established in the literature and include contradicting inferences. For example, Bruininx et al.^67^ reported that morphometric measurements (villus length, crypt depth, V:C ratio) were not affected by pre-weaning (commercial) creep feed consumption. On the other hand, a recent study^47^ reported morphological changes in the intestinal mucosa (thicker and extended villi in the jejunum) of piglets that were separated from their mother after 48 h and fed with milk replacer diet, supplemented with or without 0.8% galacto-oligosaccharides for 26 days. In the present study, we detected moderate changes in the mucosal architecture, which were only apparent in jejunal V:C ratio of early-fed piglets. Remarkably, previous findings have reported reduced villus length, increased crypt depth and (corresponding) decreased V:C ratio over time in un-weaned piglets^72,80^, thereby supporting an “accelerated maturation” of the mucosal architecture in EF piglets in this study. Likewise, it has been reported that colonic epithelial proliferation and crypt depth increase post-weaning^81^. In the present study, we used PCNA immune-histochemistry (a universal nuclear marker of proliferative cells) to assess the epithelial proliferation in jejunum and colon, showing that early-fed piglets had an increased colonic proliferation, although this difference was not reflected in the coinciding increase of colonic crypt depth. This observation would also support accelerated maturation of mucosal development by early feeding of fibrous feed. However, though both V:C ratio and colonic PCNA staining were considered to have a large effect size (Cohen’s d > 0.8), the actual fold changes in the V:C ratio (0.89) and colonic PCNA staining (1.13) were relatively small. Taken together, these are intriguing observations that would support accelerated maturation, but their actual physiological relevance would require further studies.

Overall, our study illustrates that early feeding with fibre-enriched feed influences the colonic microbiota composition, increases microbial fermentation products in the colon and modulates intestinal development at weaning. Importantly, the EF-associated changes in colonic microbial signatures were concluded to be strongly associated with the amount of eating of a piglet, which corresponded with increased weights of the small intestine and total gastro-intestinal tract (with or without digesta). Although the estimated feed intake was relatively low and highly variable among EF piglets in this study, eating behaviour quantification and the classification of individual piglets into good, moderate and bad eaters enabled a reliable and consistent evaluation of eating-behaviour consequences in piglets at an individual level.

## Methods

### Animals and experimental design

All experiments and methods were performed in accordance with relevant guidelines and regulations. The Animal Care and Use committee of Wageningen University & Research (Wageningen, The Netherlands) approved the protocol of the experiment (AVD104002016515). The protocol is in accordance with the Dutch law on animal experimentation, which complies with the European Directive 2010/63/EU on the protection of animals used for scientific purposes. The experiment was carried out in compliance with the ARRIVE guidelines (http://www.nc3rs.org.uk/page.asp?id=1357). The experiment was conducted with 12 multiparous Topigs-20 sows (range parity: 3–5), housed and inseminated at research facility Carus (Wageningen University & Research, The Netherlands). Within two days after birth, the litter size was set to a maximum of 14 piglets per litter (Tempo × Topigs-20) with no cross-fostering. The new-born piglets were cohoused with their respective sows and littermates until weaning. They received ear tags for individual identification and an iron injection, standard to pig husbandry practice. The litters were divided into two experimental groups, early-fed or EF group (n = 6) and control or CON group (n = 6) based on sow’s parity, farrowing date, body weight (of the litter at birth and 2 days of age) and genetic background. From 2 days onwards, piglets belonging to the EF group were given the opportunity to forage on customised mixed-fibre feed (Supplementary Table 1) ad libitum in addition to suckling sow’s milk whereas the CON group nursed on sow’s milk only. Due to the difference of a few days in birth-dates between litters, the same variation was evident in the actual weaning age, which was on average 29.3 ± 1.4 days of age (for details see Supplementary Table 2). For reasons of clarity, we will consistently use 29 days, as the age of weaning in the rest of the manuscript. Briefly, the diet contained 26% non-starch polysaccharides including sugarbeet pulp (4%), oat hulls (4%), inulin (4%), galacto-oligosaccharides (5%) and high amylose maize starch (4%) as fibrous ingredients. Additional details about the housing and management have been described previously^53^.

### Eating behaviour by video observation

The eating behaviour of piglets was assessed by means of video recordings, as described previously^50^. Briefly, eating frequency of individual EF piglet (identified by back numbers) was determined daily (2 days of age till weaning) from 07:00 to 19:00 h via video observations as an estimate for pre-weaning solid feed intake. From the video observations, the amount of time spent eating or “eating time” was evaluated. When an EF piglet placed its snout into the trough for a minimum of 5 s (s), the behaviour was scored as eating^52,82^. Daily/weekly eating activity per piglet was (semi-) quantified by summing the (minimum) number of seconds spent eating from 2 days of age to weaning. Of note, the eating scores were taken as an “estimated quantification” of the amount of eating per piglet. Furthermore, pre-weaning feed intake (in EF-litters) was measured between day 2–15, 15–21, and 21–30 after birth, as previously reported^53^, and we found a strong positive relationship between feed intake (measured as grams of feed consumed per litter) and ‘eating time’ (observed per piglet, and summed up per litter) on litter level (*r* = 0.87, *P* < 0.0001; Supplementary Fig. 1A). Feed wastage was kept to a minimum by placing the feeders on the solid floor in the farrowing pens (see for a picture Fig. 1 in^53^). If any, feed remains on the floor were collected.

### Intestinal microbiota sampling and microbiota metataxonomic analysis

At the end of the suckling period (just before weaning), a subset of piglets was sacrificed (n = 28; n = 14 per treatment, seven males and seven females), distributed over two consecutive sampling days (Supplementary Table 2). Piglets were euthanised by intravenous injection of 20% sodium pentobarbital (EUTHASOL, 500 mg/mL, AST Farma B.V., Oudewater, The Netherlands)^83^. The selection of sacrificed piglets were made by the following criteria: (a) no antibiotic treatment (b) close to mean body weight of the litter (c) close to average weight of the treatment group (d) one to three piglets per litter (e) equal male to female ratio. For each piglet, the gastrointestinal tract was removed from the abdominal cavity and dissected immediately to collect 20 cm of intestinal segments from different intestinal locations within 25 min after sacrifice, i.e., jejunum (1.5 m from duodenal-jejunal flexure), ileum (50 cm upstream from ileocaecal valve) and colon (mid-spiral colon) tissue. Luminal contents were collected under aseptic conditions from the intestinal tissues segments, immediately frozen in liquid nitrogen and stored at − 80 °C until further processing.

Approximately 300 mg of luminal contents (wet weight) from jejunum, ileum and colon samples was used for microbial DNA extraction. Total genomic DNA was extracted by the repeated bead beating method^84^ using QIAamp DNA Stool Mini Kit (Qiagen, Hilden, Germany) according to manufacturer’s instructions. The quality and quantity of extracted DNA was checked by gel electrophoresis and Nanodrop DeNovix DS-11 Spectrophotometer (DeNovix Inc., Wilmington, DE USA) respectively. The V3–V4 region of 16S rRNA gene was sequenced and the raw reads were processed in CLC Genomics Workbench version 11 (CLC bio, Arhus, Denmark) as described previously^50^. Briefly, the PCR amplified 16S rRNA gene was purified, extended by adaptors prior to sequencing using the Illumina MiSeq system (BaseClear BV, Leiden, The Netherlands) which generated FASTAQ sequence files, which were subjected to a BaseClear in-house quality control and filtering protocol. Subsequently, the CLC pipeline was utilised for merging the paired-end reads into one high quality representative sequence, primer and quality trimming and binning the sequences into operational taxonomic unit (OTUs) at 97% identity threshold using SILVA database v132 (released on Dec 13, 2017)^85^. To evaluate alpha and beta diversity indices, OTUs were rarefied to minimum library size (11,000 reads) attaining even sequencing depth between samples. Alpha diversity was evaluated using microbial species richness (Chao1 bias corrected) and evenness (Shannon) indicators. Relationship between microbial groups and intestinal location or treatment groups was determined by principal component analysis (PCA; unsupervised), partial redundancy analysis (pRDA; supervised) and redundancy analysis (RDA; supervised) using CANOCO 5 (Microcomputer Power, Ithaca, NY, USA), according to accompanying instructions^86^. Statistical significance was evaluated by Monte Carlo permutation procedure (MCPP) with 499 permutations. Effect of variables such as gender and sampling day were evaluated by RDA and pRDA separately, however due to their non-significant impact on microbiota, they were excluded as co-variates in further analyses. The discriminative microbial families (identified in RDA analysis of colon microbiota) were visualised in a heat map of microbial relative abundance to assess consistency of the EF treatment at individual animal level. Heat maps were constructed by hierarchical clustering of microbial groups (selected from Redundancy analysis; microbial genera below 0.01% relative abundance in less than 10% of individual samples were not included) in Perseus software^87^, where relative abundance values were log2 transformed and subsequently normalized by z-score transformation. Euclidean distance was utilized to measure the distance and clustering was conducted using the average linkage method. The online tool “MicrobiomeAnalyst”^88^ for comprehensive statistical, visual, and meta-analysis of microbiome data was also used for detecting microbial taxa which were differentially abundant among different locations/treatments. Low abundance OTUs were removed, where OTUs with less than two counts in < 10% of the samples. The OTU table was rarefied to minimum library size and transformed using trimmed mean of M-values (TMM) which was used to evaluate differentially abundant taxa (“Classical Univariate analysis” with multiple correction). To assess the beta diversity (Bray Curtis distance) between intestinal segments, PERMANOVA test was performed in MicrobiomeAnalyst.

### pH and SCFA measurement

After euthanasia, the gastro-intestinal tract was removed and the contents of the stomach, the entire small intestine, caecum and colon were collected by gently squeezing the digesta from the different parts of the intestine. Immediately after sampling, the pH was recorded by inserting a pH electrode (pH 300, HANNA Instrument, Padova, Italy) in homogenized digesta.

SCFAs (acetic, propionic, butyric acid) along with succinic acid and lactic acid, were quantified in colon digesta samples using an Ultimate 3000 HPLC equipped with an autosampler, a RI-101 refractive index detector (Shodex, Kawasaki, Japan), and an ion-exclusion Aminex HPX-87 H column (7.8 mm × 300 mm) with a guard column (Bio-Rad, Hercules, CA, USA). Samples weighing ~ 200 mg were taken in a 2 mL eppendorf tube, filled with milliQ water to have a final weight of 1 g, vortexed followed by centrifugation (10 min, 30,000 × *g*). The supernatant (10 uL) was injected onto the column and eluted with 5 mM H2SO4 at a flow rate of 0.6 mL/min at 65 °C oven temperature. Calibration curves of each acid were prepared in a range of 0.01–1 mg/mL. Chromeleon 7.1 software (Dionex, Sunnyvale, CA, USA) was used for data analysis.

### Macroscopic organ and intestinal parameters

During sacrifice, intestinal organ weights and lengths were determined. Weights of the stomach, small intestine, caecum, and colon (full and empty), as well as length of the small intestine and large intestine (caecum plus colon) were recorded for each piglet. Empty weights of the intestinal segments were determined after removal of digesta by gently squeezing the intestine, followed by rinsing of the intestine in saline solution and removal of excess rinsing fluids using paper towels.

In addition, adrenal gland, gallbladder, pancreas, spleen, and liver were also weighed. The statistical analyses were performed with linear (MIXED) mixed models in the statistical software SAS 9.4 (SAS Institute Inc., Cary, NC, USA). Organ measurements were compared with a model including treatment (EF vs. CON) as a fixed factor and using body weight at sacrifice as the covariate in the model; untransformed data are presented as means ± SEM, differences at *P* < 0.05 were considered statistically significant and differences at 0.05 ≤ *P* < 0.10 were considered as trend. For correlation analysis with the other measurements in the study, the organ-weight and -size values were converted to body-weight-normalized values by dividing absolute values by the scaled “body weight factor” (body weight of an animal divided by the smallest body weight in the group; scaled between 1 and 1.9) to obtain “normalized organ-weight and -size values” per animal.

### Histological morphometric measurements and immunohistochemical staining of intestinal proliferating cells

At weaning, piglets were sacrificed and their intestinal sections (about 2 cm) from proximal jejunum and mid colon (n = 16, 8 per treatment) were fixed with 4% paraformaldehyde (PFA), and then dehydrated and embedded in paraffin blocks. 5 µm sections were cut with a Accu-Cut SRM 200 Rotary Microtome (Sakura Finetek Europe B.V., Alphen aan de Rijn, The Netherlands), deparaffinized, hydrated and stained with Haematoxylin–eosin (H&E). Slides were examined using a Leica DM6 B microscope (Leica Microsystems Ltd. CH9435 Heerbrugg) and images (5 × magnification) were processed with LAS X software (Leica Microsystems Inc., Buffalo Grove, IL, USA). Intestinal (histo-)morphometric parameters villus length and crypt depth (μm) were measured from jejunal sections and crypt depth was measured from colonic tissue sections. These parameters were measured on 90 well-formed villi and their corresponding crypts per animal (n = 8 per group; three intestinal sections per animal). In jejunum, the villus length was defined from the tip of a villus to their base and the crypt depth was measured as a distance from the base of the villus (i.e., villus-crypt transition) to muscularis mucosa. Subsequently, the ratio of the villus length to crypt depth (V:C) was calculated.

For immunohistochemical staining of proliferating cells in intestinal tissues, 5 µm sections were deparaffinized, rehydrated and treated for antigen retrieval in citrate buffer (pH 6.0) at 95 °C for 20 min, followed by cooling in tris-buffered saline and tween 20 (TBSt) buffer for 20 min at room temperature (RT). Non-specific staining were blocked using 10% goat serum for 30 min at RT. To detect proliferating cells, sections were incubated with primary antibody (anti-PCNA antibody, PC10 mouse anti-rat IgG2a monoclonal antibody, Merck-millipore, Darmstadt, Germany, MAB424R; 1:200) overnight at 4 °C, followed by TBSt washing and incubation with secondary antibody (Goat anti-Mouse IgG (H + L) Superclonal Secondary Antibody, Alexa Fluor 555, ThermoFisher Scientific, Waltham, Massachusetts, USA; 1:300) for 1 h at RT. Nuclei were stained with Hoechst 33,342 Solution (Invitrogen, ThermoFisher; 1:1000 dilution). Negative controls (TBSt replacing primary antibody) were also included during the staining procedures. For quantification of proliferative cells, 10 high quality 16bit grayscale images were captured per animal for both locations (80 representative images//treatment group/location) at 20 × magnification using Leica DM6b microscope fitted with appropriate fluorescence filters along with their corresponding nuclei images. Image analysis was performed using Cell Profiler 3.1.8 (Broad Institute, Cambridge Massachusetts USA; www.cellprofiler.org) and FCS Express 6 Flow plus Image (De Novo Software, CA, USA, www.denovosoftware.com), as described in Supplementary file 1. PCNA is a nuclear stain and thus the number of proliferating cells was normalized by the total number of Hoechst positive nuclei in an image, to obtain ratio of PCNA:Hoescht identified nuclei/cells.

### Association of eating scores in EF piglets with multiple readouts

We first investigated the impact of early feeding on microbiota composition (colon), pH (caecum/colon), SCFAs (colon), macroscopic and microscopic intestinal measurements. Subsequently, we evaluated whether these parameters are quantitatively associated with individualised quantification estimates of eating behaviour. To evaluate the relationship between eating behaviour and other measured parameters, a non-parametric spearman correlation matrix was calculated using GraphPad Software 8.1.1 (California, USA).

### Statistical analyses

Data analyses were performed in Graph Pad Prism 8.1.1 (GraphPad Software, California USA). Normality of data (Shapiro–Wilk test) and statistical differences were checked with a limit of significance set at *P* < 0.05. Comparative analysis of the diversity indices, pH, SCFA concentrations, histological morphometric measurements and proliferating cells were performed by Mann Whitney U-test (for non-parametric) or Student's t-test (for parametric).

## Supplementary Information

Supplementary Information 1.

Supplementary Information 2.

## Data Availability

Raw sequences can be found on SRA-NCBI (Sequence Read Archive-National Center for Biotechnology Information) database under the SRA accession number PRJNA687128.
