# Supplementary material for: Impact of early-life feeding on local intestinal microbiota and digestive system development in piglets
Source: Sci Rep. 2021 Feb 18;11:4213. doi: 10.1038/s41598-021-83756-2 (PMC7892833; doi:10.1038/s41598-021-83756-2)
Supplement: Supplementary file 1 — Supplementary Information 1. [file 41598_2021_83756_MOESM1_ESM.pdf]

**Supplementary information for:**

# Impact of early-life feeding on local intestinal microbiota and digestive system development in piglets

R. Choudhury[1#], A. Middelkoop[2#], J.G. de Souza[1], L.A. van Veen[1], W.J.J. Gerrits[3], B. Kemp[2], J.E. Bolhuis[2], M. Kleerebezem[1]*

[1]*Host-Microbe Interactomics Group, Department of Animal Sciences, Wageningen University & Research, P.O. Box 338, 6700 AH Wageningen, The Netherlands*

[2]*Adaptation Physiology Group, Department of Animal Sciences, Wageningen University & Research, P.O. Box 338, 6700 AH Wageningen, The Netherlands*

[3]*Animal Nutrition Group, Department of Animal Sciences, Wageningen University & Research, P.O. Box 338, 6700 AH Wageningen, The Netherlands*

**Supplementary table 1:** Ingredients and calculated nutrient composition of the pre-weaning fibrous feed[1].

| | **Pre-weaning feed** |
|---|---|
| **Ingredients, %** | |
| Wheat | 21.9 |
| Barley | 15 |
| Maize | 15 |
| Soy protein concentrate | 7 |
| Soybeans (heat treated) | 5 |
| **Galacto-oligosaccharides[2]** | **5** |
| Potato protein | 4 |
| **Sugar beet pulp** | **4** |
| **Oat hulls** | **4** |
| **Inulin[3]** | **4** |
| **Resistant starch[4]** | **4** |
| Soybean oil | 3 |
| Blood meal (spray dried) | 2 |
| Dicalcium phosphate | 1.7 |
| Sucrose | 1.5 |
| Calcium carbonate | 1.0 |
| Sodium chloride | 0.5 |
| Premix[5] | 0.5 |
| Potassium bicarbonate | 0.3 |
| L-lysine hydrochloride | 0.3 |
| DL-methionine | 0.2 |
| L-threonine | 0.04 |
| L-tryptophan | 0.04 |
| | |
| **Calculated nutrient composition[6], g/Kg** | |
| Dry matter | 891 |
| Starch | 290 |
| NSP[7] | 261 |
| Crude protein | 195 |
| Crude fat | 61 |
| Crude fibre | 44 |
| Crude ash | 57 |
| Calcium | 9.1 |
| Phosphorus | 6.1 |
| Sodium | 2.2 |
| Standardized ileal digestible lysine | 11.9 |
| Standardized ileal digestible methionine | 4.8 |
| Standardized ileal digestible threonine | 7.1 |
| Standardized ileal digestible tryptophan | 2.4 |
| Net energy, MJ/kg | 11.8 |

[1] Feed was mixed by Research Diet Services (Wijk bij Duurstede, The Netherlands), and extruded using a co-rotating double screw extruder (M.P.F. 50, Baker Perkins, Peterborough, United Kingdom).
[2] Source: Vivinal® GOS powder (Friesland Campina, Amersfoort, The Netherlands) containing 69% galacto-oligosaccharides.
[3] Source: Prebiofeed 95 inulin powder (Cosucra group, Belgium) containing 85% inulin.
[4] Source: AmyloGel® Native Starches (Cargill, Wayzata, USA) derived from high amylose maize with 75% amylose content.
[5] Vitamin and mineral premix (per kg of feed): vitamin A: 10000 IU, vitamin D3: 2000 IU, vitamin E: 40 mg, vitamin K: 1.5 mg, vitamin B1: 1 mg, vitamin B2: 4 mg, vitamin B6: 1.5 mg, vitamin B12: 0.02 mg, niacin: 30 mg, D-pantothenic acid: 15 mg, choline chloride: 150 mg, folate: 0.4 mg, biotin: 0.05 mg, iron: 100 mg, copper: 20 mg, manganese: 30 mg, zinc: 70 mg, iodine: 0.7 mg, selenium: 0.25 mg, anti-oxidant: 125 mg.
[6] According to CVB (2007), nutrients are presented in g/kg dry matter, except for dry matter (g/kg) and net energy (MJ/kg).
[7] Non-starch polysaccharides: Calculated as the difference between dry matter and the sum of starch, sugars, crude protein, crude fat, and crude ash.

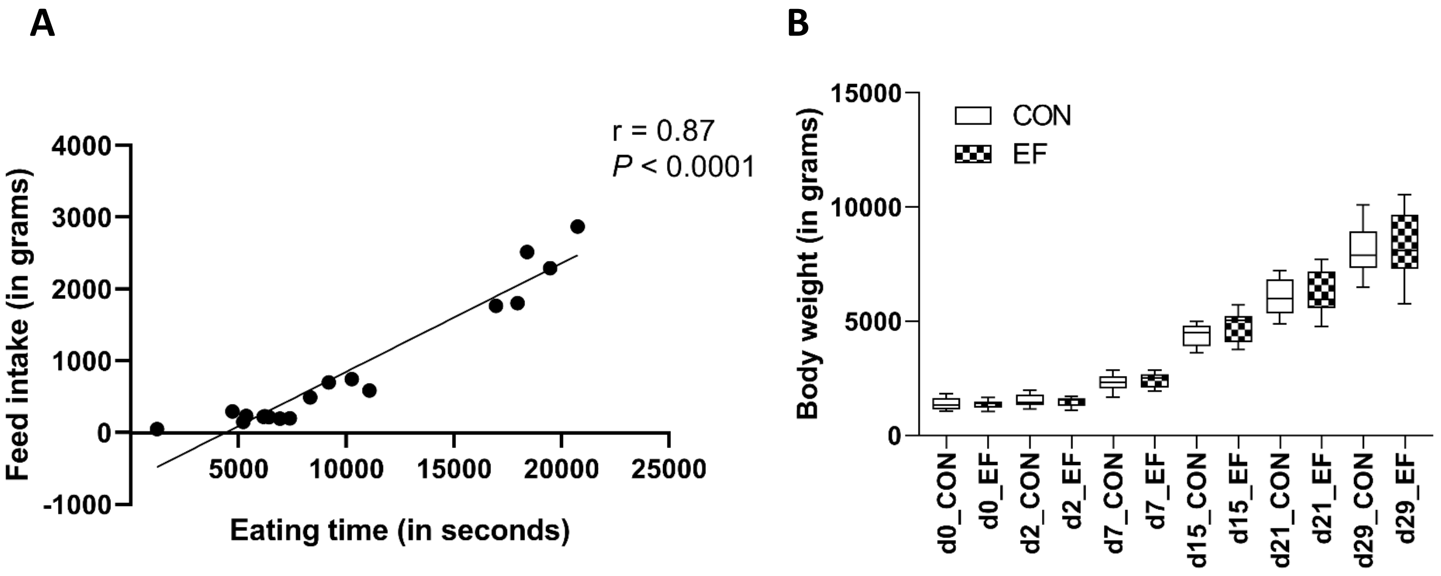


**Supplementary figure 1: (A)** Spearman correlation of feed intake (measured) and eating time (observed) on litter level, indicating a strong positive relationship between them in early-fed (EF) piglets. **(B)** Pre-weaning body weight development of control (CON) and EF groups at birth (d0), 2 (d2), 7 (d7), 15 (d15), 21 (d21) days after birth and at weaning (d29).

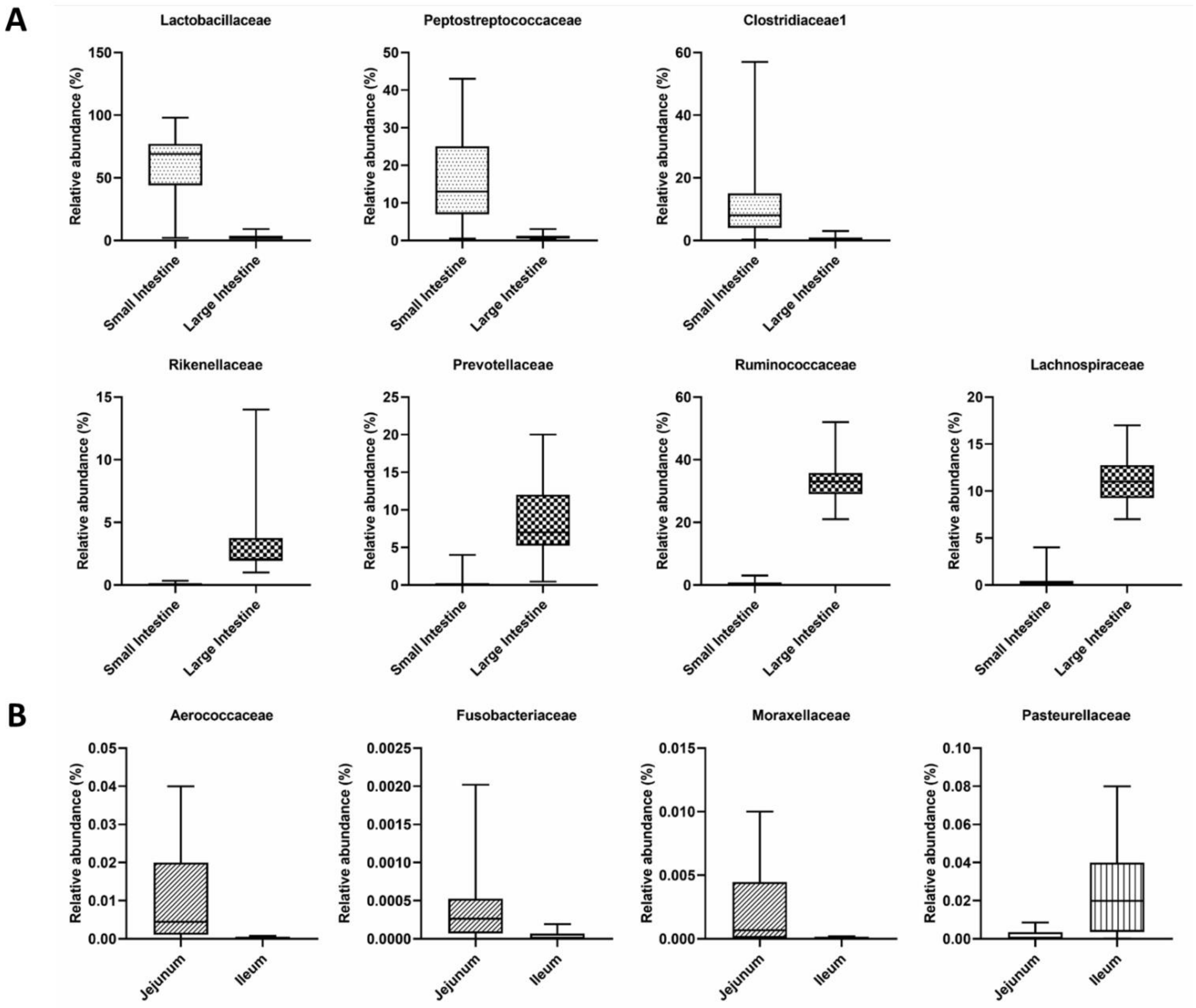


**Supplementary figure 2:** Box plots of the relative abundance of microbial families at different intestinal locations. **(A)** Comparing small and large intestine. **(B)** Comparing jejunum and ileum. Representative families which have significant (FDR corrected *P* value < 0.0001) differential relative abundance are shown in this figure.

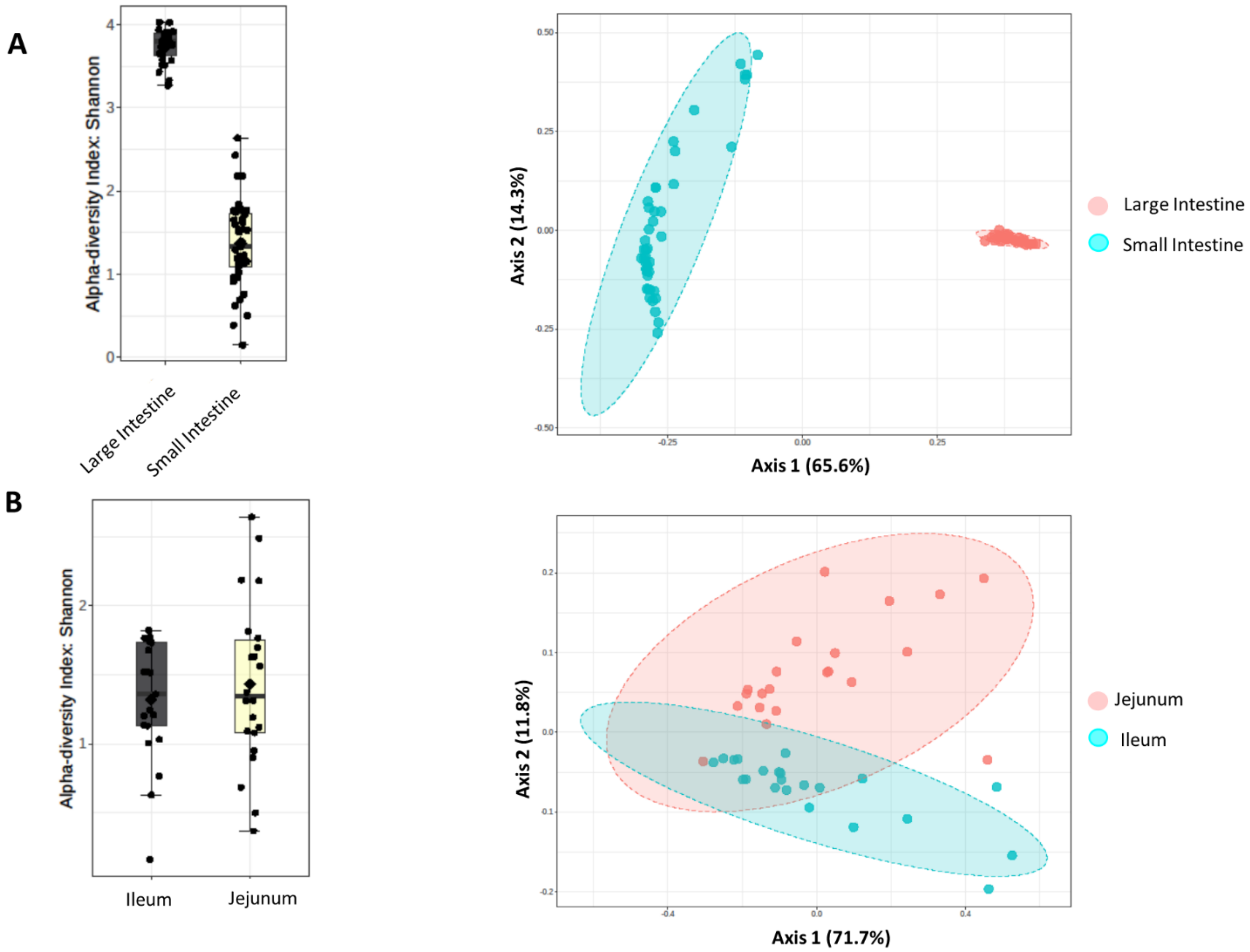


**Supplementary figure 3:** Comparison of diversity metrices among different intestinal locations at genus level. **(A)** Comparison between small and large intestine using alpha diversity (Shannon: $P < 0.0001$) and beta diversity (PERMANOVA of Bray Curtis distance as depicted in PCoA 2D plot; $P < 0.001$). **(B)** Comparison between small intestinal locations jejunum and ileum using alpha diversity metrices (Chao1: $P = 8.1931e\text{-}071$; Shannon: $P = 0.49$) and beta diversity (PERMANOVA of Bray Curtis distance as depicted in PCoA 2D plot; $P < 0.052$). All diversity comparisons were performed after rarefying the reads to minimum library size.

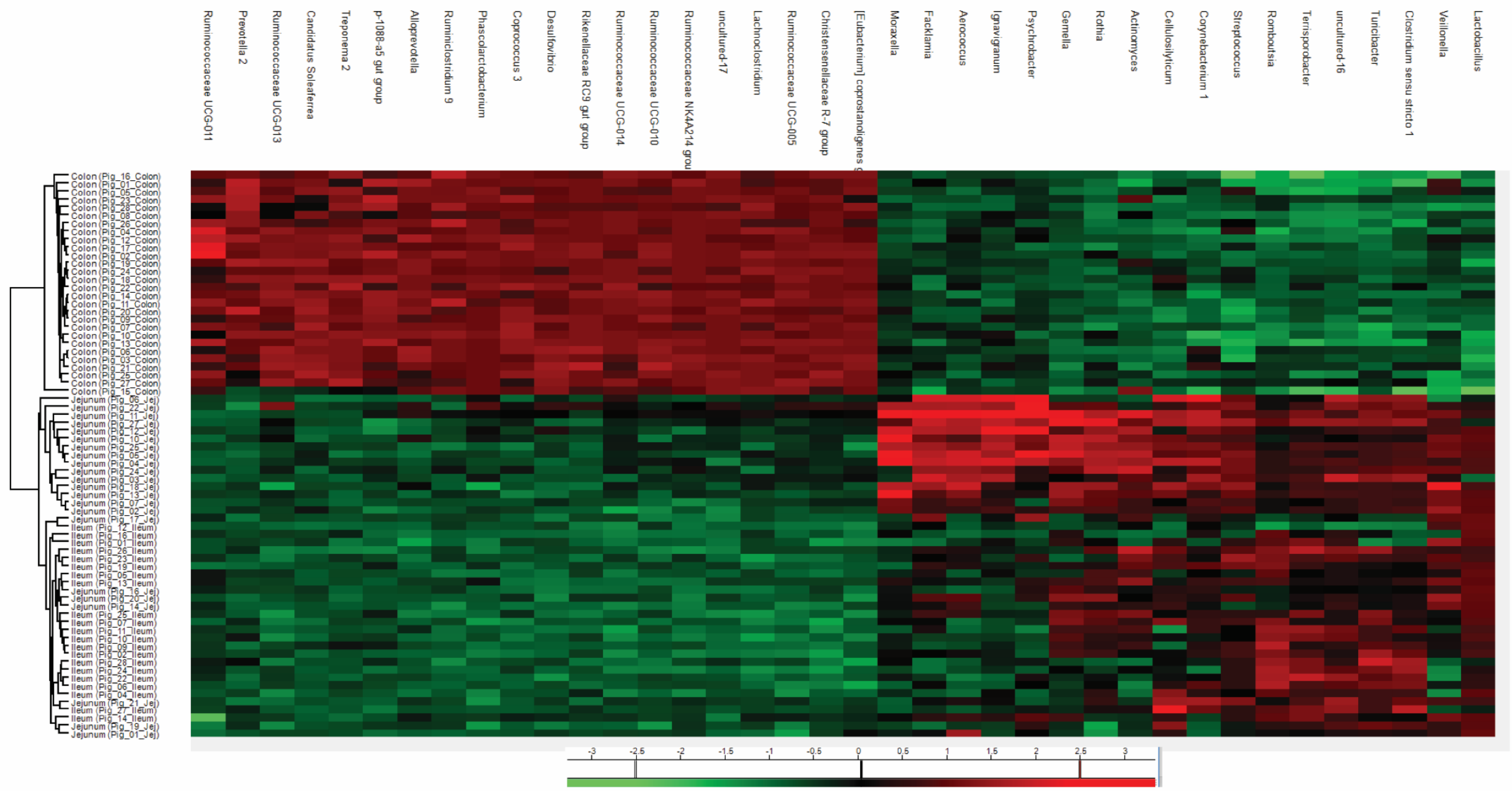


**Supplementary figure 4:** Heat map (of individual animals) showing relative abundance of discriminative bacterial groups as identified in redundancy analysis as a function of "intestinal location" at genus level.

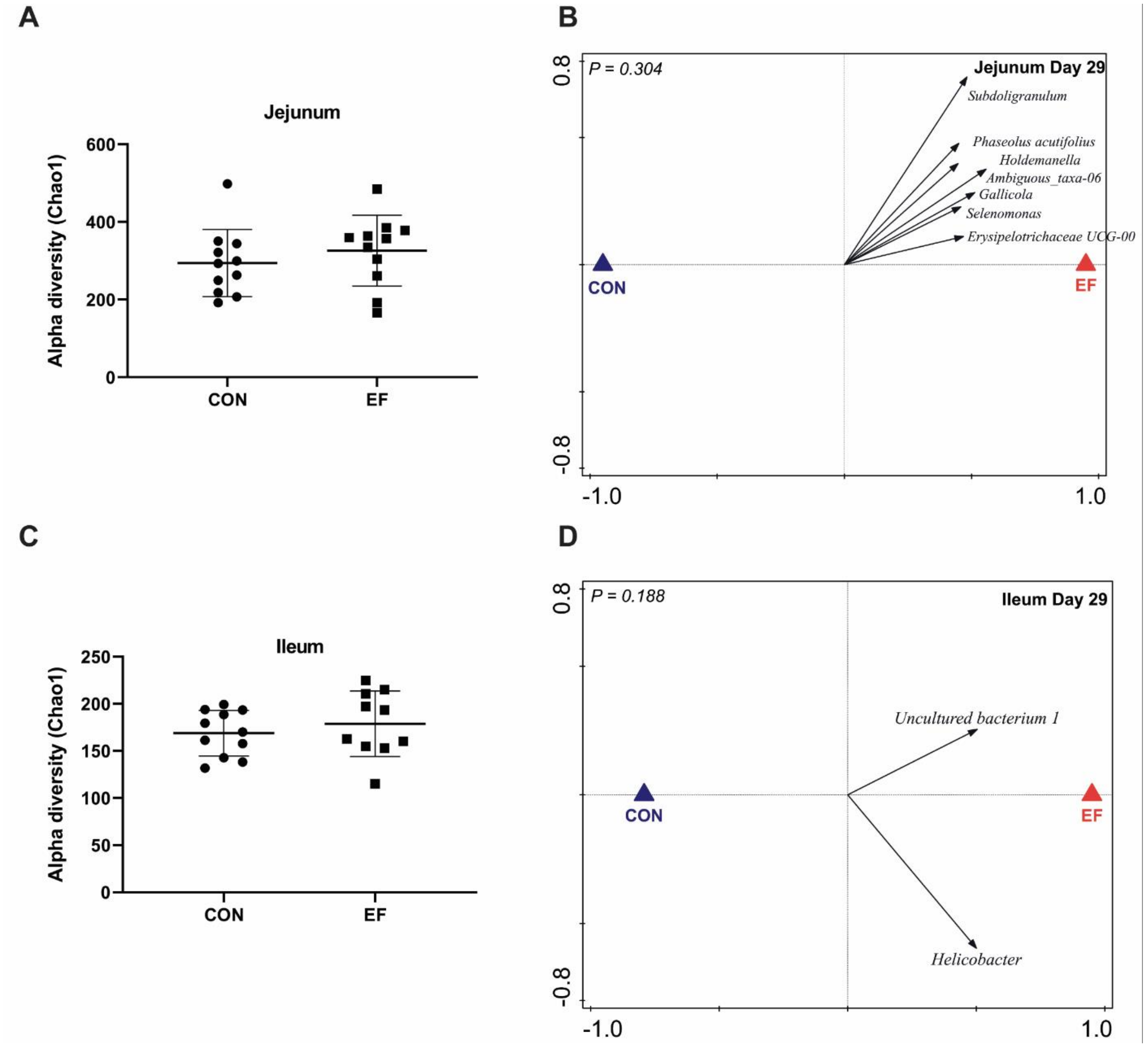


**Supplementary figure 5: Jejunal and Ileal microbiota.** Alpha diversity (Chao1 bias corrected) comparison between early-fed (EF) and control (CON) group in (**A**) jejunum and (**C**) Ileum. Redundancy analysis at genus level in **(B)** jejunum (Explained variation = 0.65%; PC1 = 5.38%, PC2 = 27.85%; *P* = 0.304) and (**D**) ileum (Explained variation = 1.27%; PC1 = 5.97%, PC2 = 18.36%; *P* = 0.188), with associated microbial groups shown (20% minimum fit on horizontal axis).

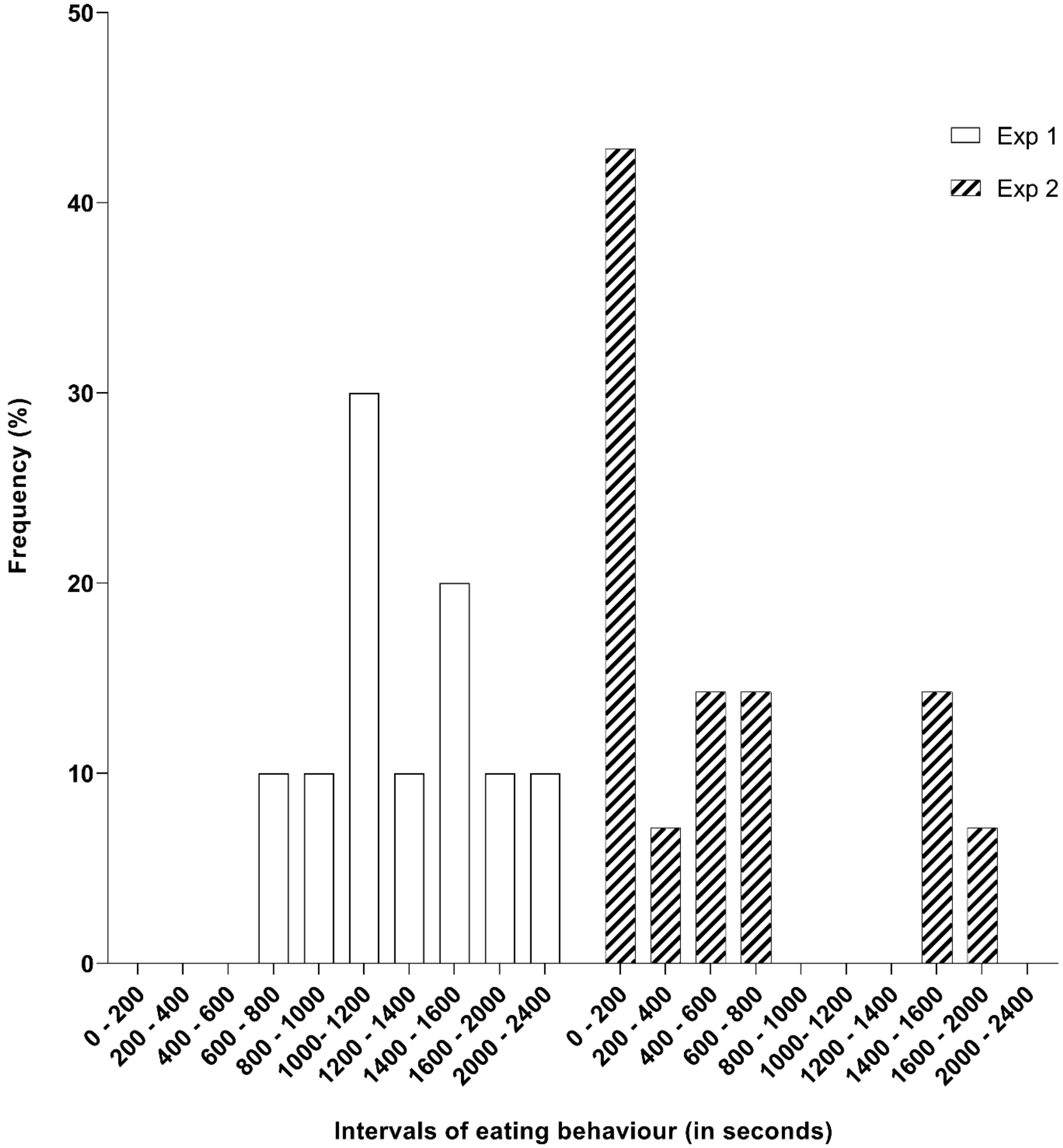


**Supplementary figure 6:** Frequency of eating scores in last two days before weaning, comparing two experiments (exp1 = previous study[50]; exp2 = this study) with respect to eating behaviour. Frequency distribution of EF piglets at various intervals (200 seconds considered per interval) of eating behaviour two days before weaning. The total eating score was also lower in the present study (3204 ± 1810 seconds) compared to the previous one (8032 ± 3387 seconds).

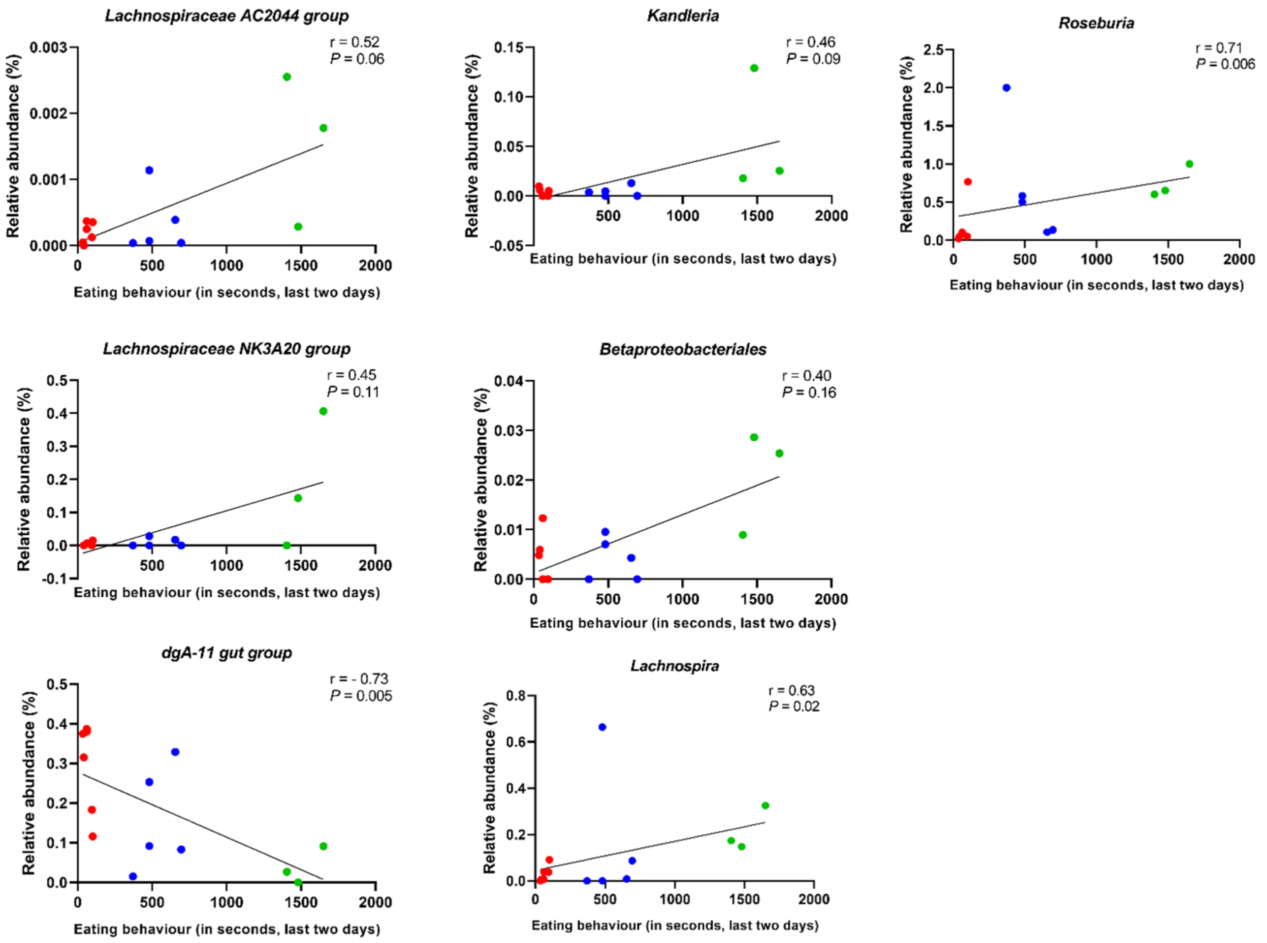


**Supplementary figure 7:** Correlation of individual microbial genera with the "classified" eating scores per individual during the last two days before weaning identified in redundancy analysis. The early-fed (EF) piglets are marked with colours of EF classification based on eating observed in the "last two days" before weaning. Green = good eaters; Blue = moderate eaters; Red = bad eaters.

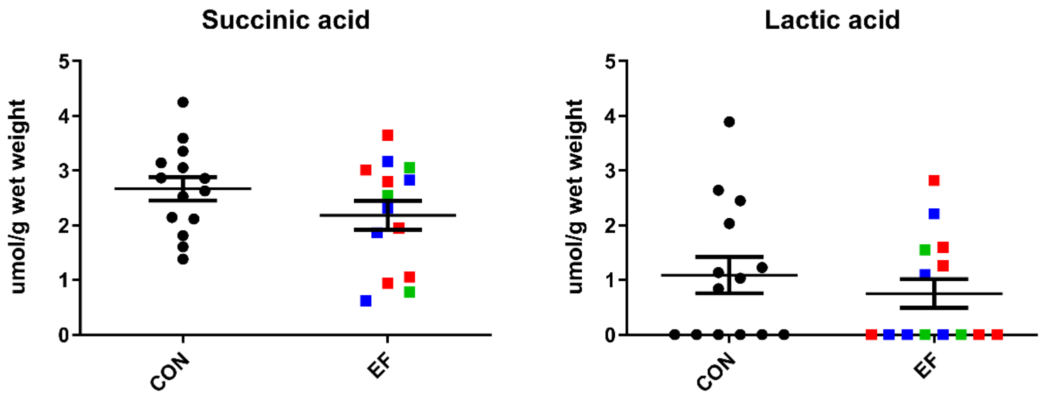


**Supplementary figure 8:** Concentrations of short chain fatty acids (SCFA) succinic and lactic acid in control (CON) and early-fed (EF) piglets. The EF piglets are marked with colours of EF classification based on eating observed in the "last two days" before weaning. Green = good eaters; Blue = moderate eaters; Red = bad eaters.

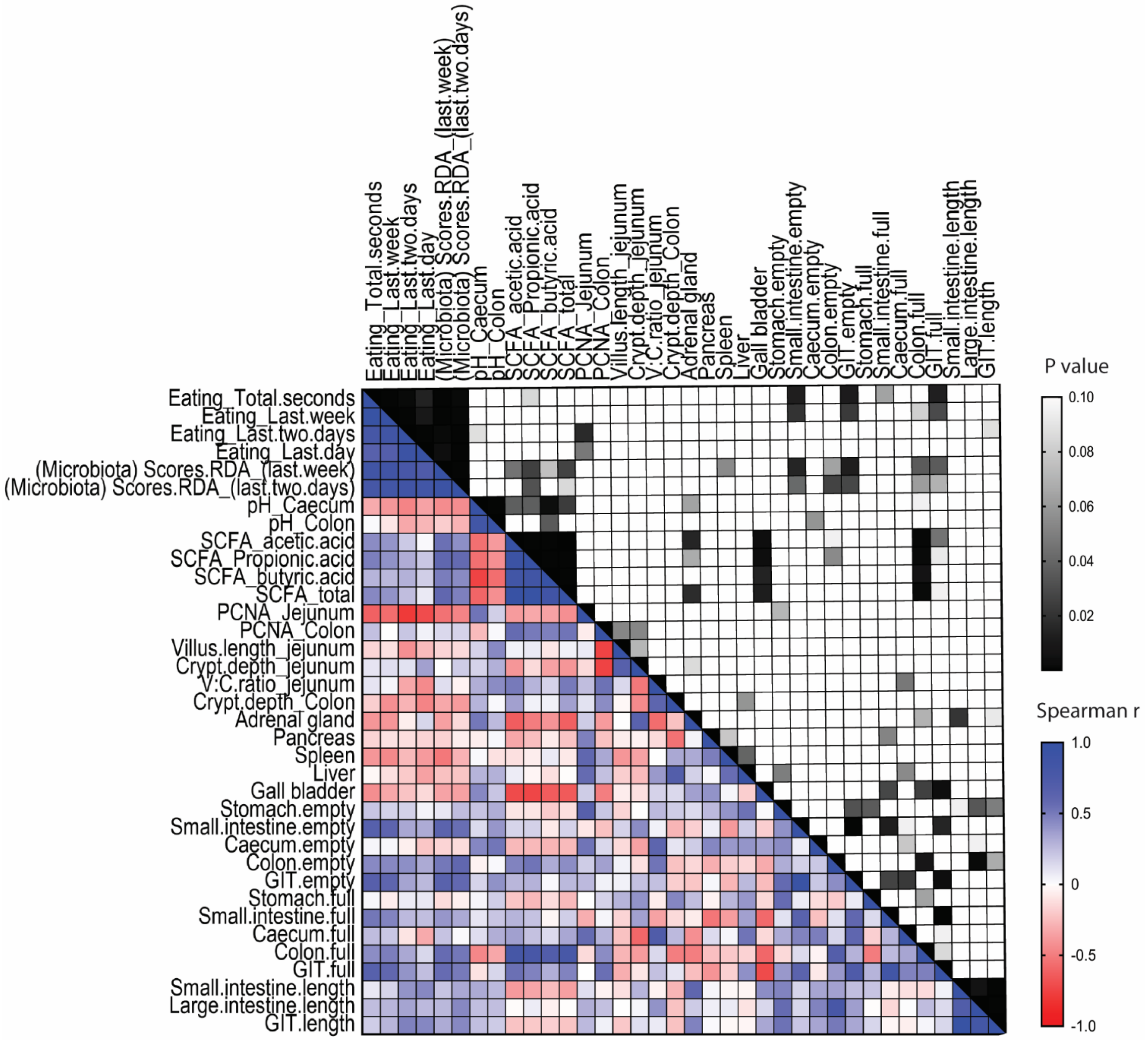


**Supplementary figure 9:** Correlation matrix (spearman) of different measured parameters of individual early-fed (EF) piglets including eating scores, microbiome RDA score, pH, SCFA, PCNA, intestinal morphometry and intestinal macroscopic measurements.

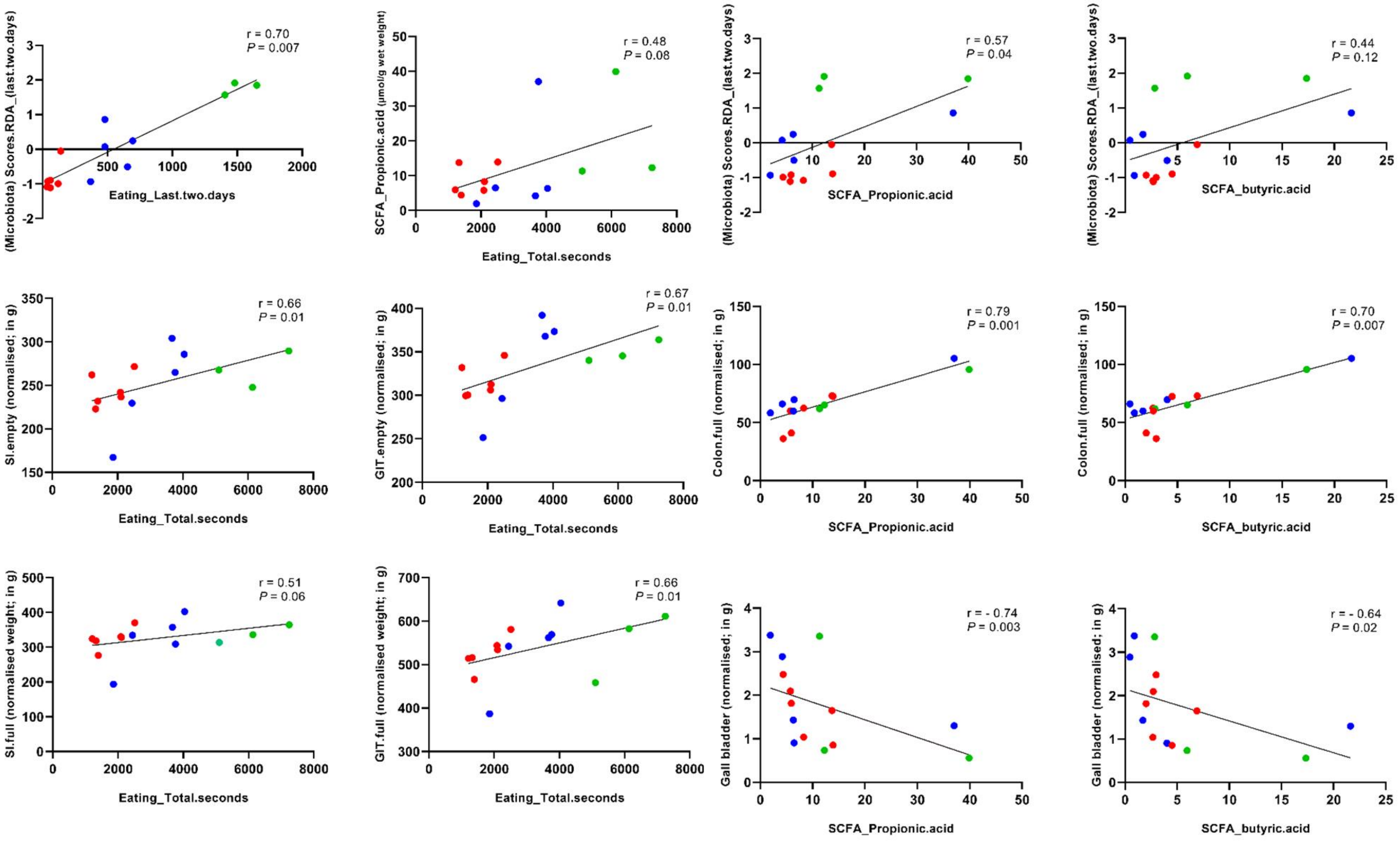


**Supplementary figure 10:** Representative parameters identified in the correlation matrix visualised in separate (spearman) correlation plots. The early-fed (EF) piglets are marked with colours of EF classification based on eating observed in the “last two days” before weaning (Green = good eaters; Blue = moderate eaters; Red = bad eaters). The piglets classified as good eaters are also the best eaters when “total seconds” are considered, but the moderate/bad eaters classification is less reliable, with two moderate piglets categorised as bad eaters in “total seconds”.

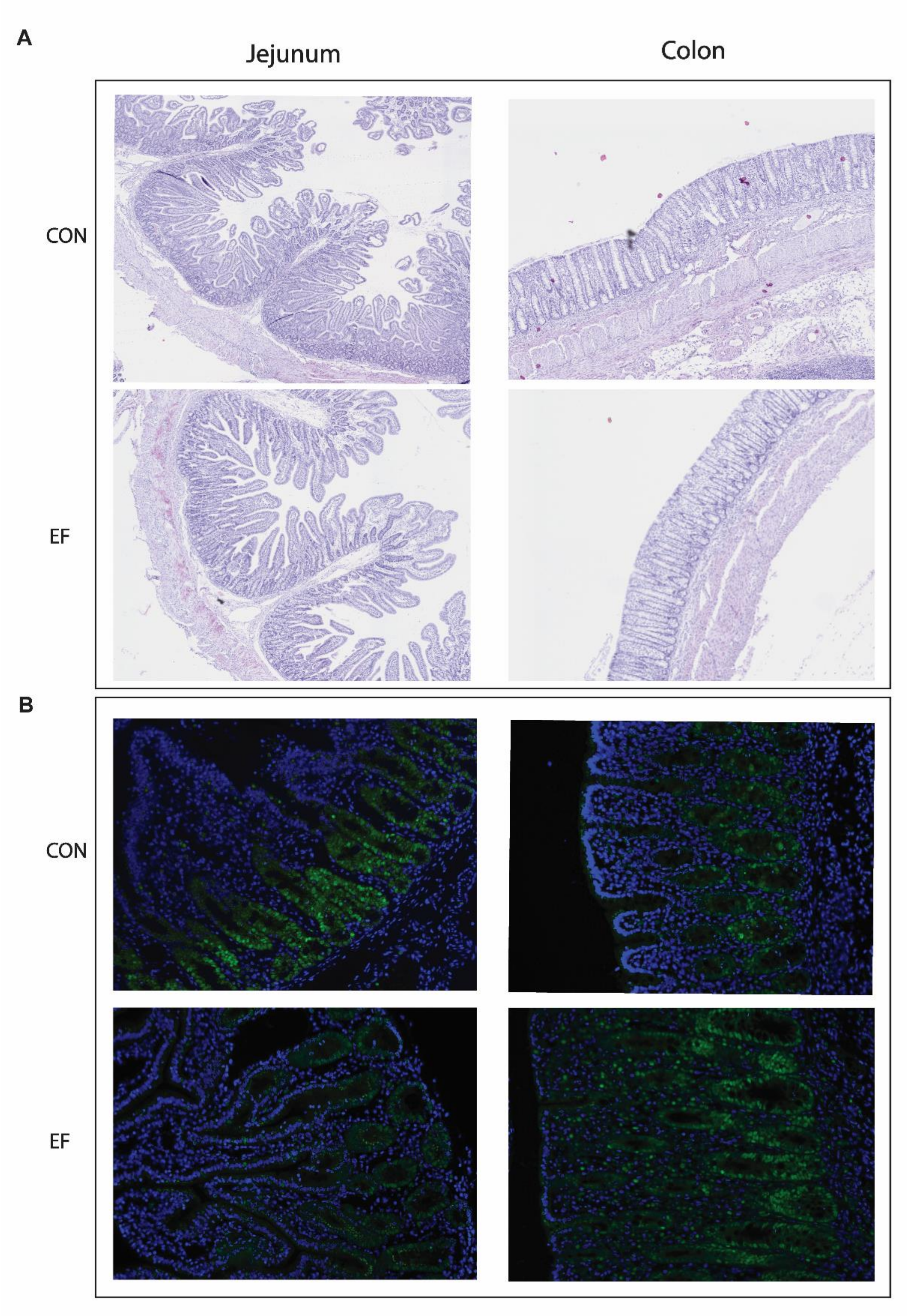


**Supplementary figure 11:** Representative images of control (CON) and early-fed (EF) groups for **(A)** intestinal morphometry and **(B)** PCNA positive proliferating cells in jejunum and colon tissue (overlay images).

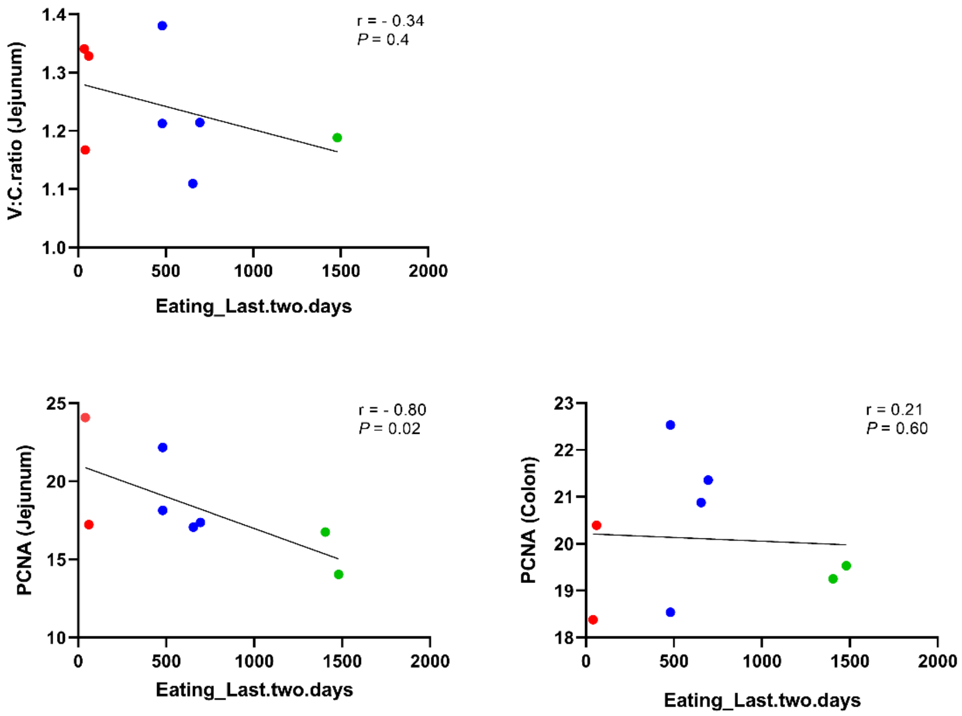


**Supplementary figure 12:** Spearman correlation of microscopic measurements (V:C ratio and PCNA) with eating score. Green = good eaters; Blue = moderate eaters; Red = bad eaters.

**Supplementary file 1: Quantitative immunostaining**

The image analysis pipeline employed Cell Profiler 3.1.8 (Broad Institute, Cambridge Massachusetts USA; www.cellprofiler.org) to identify PCNA/Hoechst positive nuclei FCS Express 6 Flow plus Image (De Novo Software, CA, USA, www.denovosoftware.com) to visualise and enumerate the identified nuclei. The raw (16-bit grayscale) images were analysed in a pipeline developed in Cell Profiler, consisting of different modules, which carry out automated mining of cellular features in every image. The Hoechst positive nuclei (primary objects) are identified in the first module with nuclei ranging between 2 to 20 pixels in diameter and applying an adaptive threshold strategy in combination with the two-classes Otsu algorithm. In the second module, PCNA positive nuclei (secondary objects) are identified by using primary objects as a reference for guided detection. The method uses the propagation algorithm (regularisation factor, $\lambda = 0.05$), which assigns (a) dividing lines between secondary objects that touch each other and (b) dividing lines between the secondary objects and the background of the image, taking into account both the distance to the nearest primary object and intensity gradients. The subsequent modules extract features such as mean intensity, size from each object and converts identified objects back into images. The output derived from Cell profiler (.cpout file), containing the raw data associated with individual objects and analysis, was used to visualise and enumerate the number of ide ntified primary and secondary objects in FCS Express 6 Plus. The size distribution, intensity of all identified objects was visualised in a histogram and three gates (or markers) were utilized to include objects (i.e., nuclei) based on size-limits (red; gate 1), minimal Hoechst intensity (blue; gate 2) and minimal intensity of PCNA staining (green; gate 3). The flow chart below depicts the gate-settings and the corresponding images that display the included nuclei in each gating step. Gate 1 (or M1) is based on size distribution with a lower bound of Median-SDV (SDV = Standard deviation)

and a upper bound of Median+3SDV, leading to inclusion of the red-stained objects (i.e., nuclei; 97 ± 0.2% of total nuclei) in the corresponding image, and exclusion of white-stained objects that mostly represent overlapping or over-exposed nuclei. Gate 2 (or M2) is based on Hoechst-staining intensity with a lower bound of Median-2SDV and a upper bound of Median+3SDV, leading to inclusion of blue-stained objects (98 ± 0.5% of the nuclei that passed gate 1). Gate 3 (M3) is based on PCNA intensity with Median+SDV and Median+10SDV as lower and upper bound, leading to inclusion of high-intensity PCNA objects (displayed as green-stained nuclei; 18 ± 3.32% of the nuclei that passed gate 2). The objects passing all three gating steps represent nuclei that are within the nucleus-size boundaries and are considered positive in Hoechst and PCNA staining. The gating values were based on the highest (visual) resemblance of the processed images (picture plot showing patterns of identified objects/nuclei in the tissue, marked by different gates) with the original microscopic overlayed-images of Hoechst and PCNA staining. From the processed images, the number of Hoechst and PCNA positive objects were obtained and the ratio of PCNA:Hoechst positive nuclei was calculated to assess the relative fraction of proliferating cells in the mucosal tissue of individual animals.

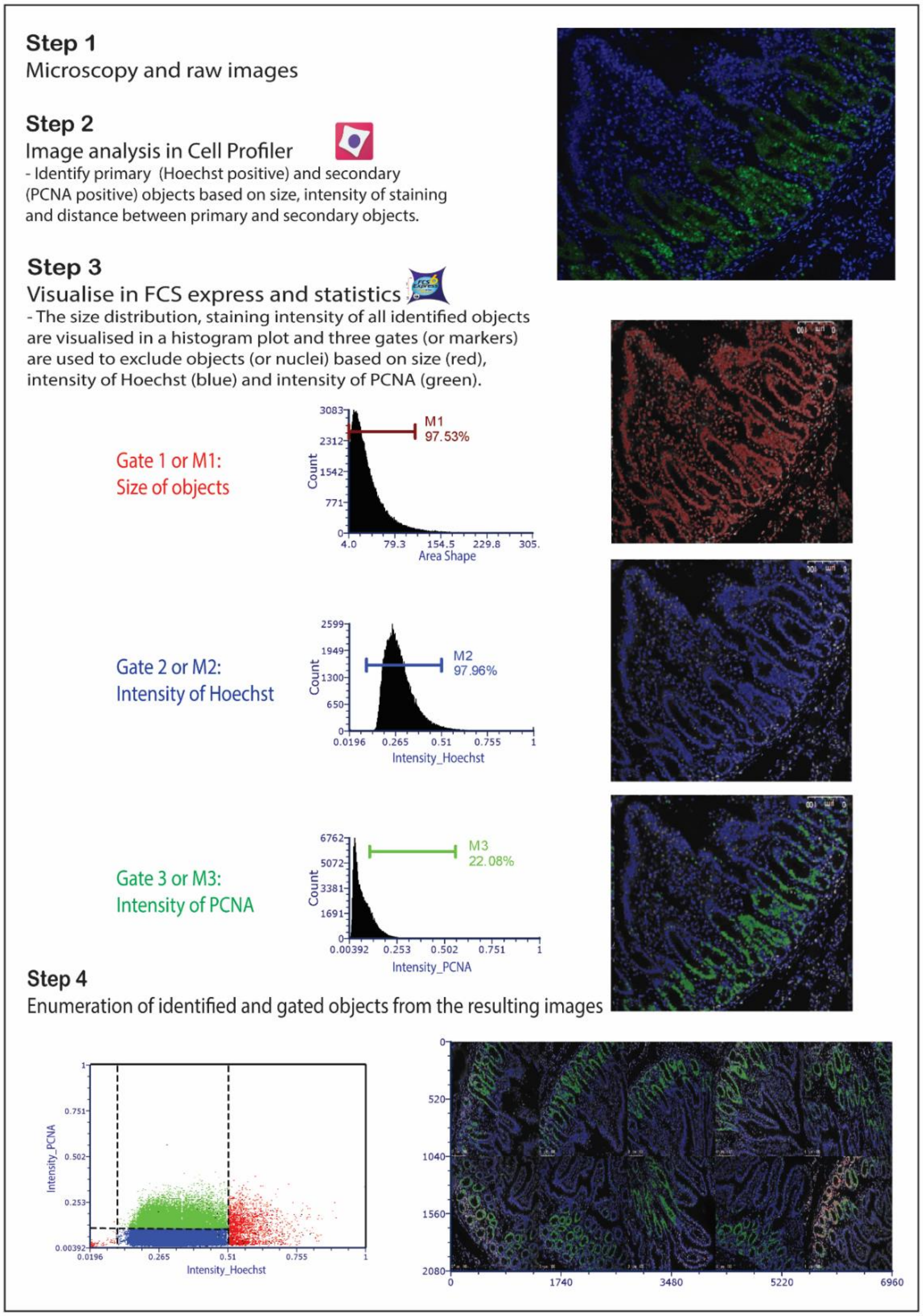


Flowchart outlining the steps for quantitative immunostaining of intestinal proliferative cells. **Step 1**: Raw microscopic image with Hoechst (blue) and PCNA (green) staining. **Step 2**: Identification of primary and secondary objects in Cell Profiler based on size, intensity of staining and distance to corresponding primary object. **Step 3**: Identified objects or nuclei were visualised and analysed in FCS Express. Three gates (or markers) were utilized to include objects (or nuclei) based on size-limits (red), minimal intensity of Hoechst staining (blue) and minimal intensity of PCNA staining (green). The intensity of Hoechst and PCNA identified objects (gated by M1, M2 and M3) is shown in a dot plot. **Step 4**: The resulting images provide the number of Hoechst and PCNA positive objects and then the ratio of PCNA:Hoechst positive nuclei was calculated.
